# Side-by-Side Comparison of Five Chelators for ^89^Zr-Labeling of Biomolecules: Investigation of Chemical/Radiochemical Properties and Complex Stability

**DOI:** 10.3390/cancers13246349

**Published:** 2021-12-17

**Authors:** Helen Damerow, Ralph Hübner, Benedikt Judmann, Ralf Schirrmacher, Björn Wängler, Gert Fricker, Carmen Wängler

**Affiliations:** 1 Biomedical Chemistry, Clinic of Radiology and Nuclear Medicine, Medical Faculty Mannheim, Heidelberg University, Theodor-Kutzer-Ufer 1-3, 68167 Mannheim, Germany; Helen.Damerow@medma.uni-heidelberg.de (H.D.); Ralph.Huebner@medma.uni-heidelberg.de (R.H.); Benedikt.Judmann@medma.uni-heidelberg.de (B.J.); 2 Molecular Imaging and Radiochemistry, Clinic of Radiology and Nuclear Medicine, Medical Faculty Mannheim, Heidelberg University, Theodor-Kutzer-Ufer 1-3, 68167 Mannheim, Germany; Bjoern.Waengler@medma.uni-heidelberg.de; 3 Department of Oncology, Division of Oncological Imaging, University of Alberta, 11560 University Avenue, Edmonton, AB T6G 1Z2, Canada; schirrma@ualberta.ca; 4 Institute of Pharmacy and Molecular Biotechnology, University of Heidelberg, Im Neuenheimer Feld 329, 69120 Heidelberg, Germany; gert.fricker@uni-hd.de

**Keywords:** ^89^Zr, DFO, CHT-36, DFO*, 3,4,3-(LI-1,2-HOPO), DOTA-GA, bioconjugation, complex inertness

## Abstract

**Simple Summary:**

The positron emitter ^89^Zr^4+^ is an important radionuclide for the preparation of radiolabeled antibodies, being applied in highly specific and sensitive positron emission tomography (PET) imaging of malignancies. The introduction of ^89^Zr^4+^ into biomolecules is performed using chelating agents, wrapping up the radiometal and preventing its release from the antibody by forming so-called complexes. Desferrioxamine B (DFO) is the clinical gold standard chelator for the preparation of ^89^Zr antibodies despite its known inability to stably encapsulate the radiometal, resulting in ^89^Zr release and associated challenges such as decreased image quality and radiation dose to healthy tissues. Therefore, several research groups have been working to develop new chelating agents able to stably encapsulate the ^89^Zr^4+^ ion. However, there are no data available directly comparing the stability of the formed ^89^Zr complexes of the most promising chelating agents developed so far. Here, we report on the comparison of five different chelators with high potential for stable complexation of ^89^Zr and determined two of them—DFO* and 3,4,3-(LI-1,2-HOPO)—to be highly interesting for the preparation of ^89^Zr-based radiolabeled agents and routine clinical application.

**Abstract:**

In this work, five different chelating agents, namely DFO, CTH-36, DFO*, 3,4,3-(LI-1,2-HOPO) and DOTA-GA, were compared with regard to the relative kinetic inertness of their corresponding ^89^Zr complexes to evaluate their potential for in vivo application and stable ^89^Zr complexation. The chelators were identically functionalized with tetrazines, enabling a fully comparable, efficient, chemoselective and biorthogonal conjugation chemistry for the modification of any complementarily derivatized biomolecules of interest. A small model peptide of clinical relevance (TCO-c(RGDfK)) was derivatized via iEDDA click reaction with the developed chelating agents (TCO = trans-cyclooctene and iEDDA = inverse electron demand Diels-Alder). The bioconjugates were labeled with ^89^Zr^4+^, and their radiochemical properties (labeling conditions and efficiency), log*D*_(7.4)_, as well as the relative kinetic inertness of the formed complexes, were compared. Furthermore, density functional theory (DFT) calculations were conducted to identify potential influences of chelator modification on complex formation and geometry. The results of the DFT studies showed—apart from the DOTA-GA derivative—no significant influence of chelator backbone functionalization or the conjugation of the chelator tetrazines by iEDDA. All tetrazines could be efficiently introduced into c(RGDfK), demonstrating the high suitability of the agents for efficient and chemoselective bioconjugation. The DFO-, CTH-36- and DFO*-modified c(RGDfK) peptides showed a high radiolabeling efficiency under mild reaction conditions and complete ^89^Zr incorporation within 1 h, yielding the ^89^Zr-labeled analogs as homogenous products. In contrast, 3,4,3-(LI-1,2-HOPO)-c(RGDfK) required considerably prolonged reaction times of 5 h for complete radiometal incorporation and yielded several different ^89^Zr-labeled species. The labeling of the DOTA-GA-modified peptide was not successful at all. Compared to [^89^Zr]Zr-DFO-, [^89^Zr]Zr-CTH-36- and [^89^Zr]Zr-DFO*-c(RGDfK), the corresponding [^89^Zr]Zr-3,4,3-(LI-1,2-HOPO) peptide showed a strongly increased lipophilicity. Finally, the relative stability of the ^89^Zr complexes against the EDTA challenge was investigated. The [^89^Zr]Zr-DFO complex showed—as expected—a low kinetic inertness. Unexpectedly, also, the [^89^Zr]Zr-CTH-36 complex demonstrated a high susceptibility against the challenge, limiting the usefulness of CTH-36 for stable ^89^Zr complexation. Only the [^89^Zr]Zr-DFO* and the [^89^Zr]Zr-3,4,3-(LI-1,2-HOPO) complexes demonstrated a high inertness, qualifying them for further comparative in vivo investigation to determine the most appropriate alternative to DFO for clinical application.

## 1. Introduction

In medical applications, whole-body imaging of malignant tissue represents a standard procedure for the diagnosis of cancer. For this purpose, different methods such as magnetic resonance imaging (MRI), computed tomography (CT) and positron emission tomography (PET) are used, each with its own specific advantages. PET, for example, offers the unique advantage of requiring such small amounts of radiotracers that target structure-specific imaging of malignant tissues becomes possible with high sensitivity. Thus, PET allows not only delineation but also the functional characterization of tumors. Furthermore, PET enables the determination of an appropriate dose of a potential endoradiotherapeutic agent and the assessment of a therapeutic response to a therapeutic agent, as well as therapy monitoring.

The radiotracers used can be based on known drugs, comprising compounds of low molecular weight, peptides and artificial functionalized nanocarriers, as well as antibodies. Antibodies have the advantage to bind to their target structure with very high specificity and binding strength, leading to an improved accumulation at the target site. Hence, radiolabeled antibodies can be used to obtain high-contrast diagnostic images that delineate the tumor with high specificity and sensitivity. Due to the relatively slow in vivo pharmacokinetics and long blood pool residence time, the physical half-life of the radionuclide used for the labeling of antibodies has to match its biological half-life. This is the reason why ^89^Zr^4+^ is mostly used for antibody labeling, as it has a physical half-life of 3.3 days, perfectly matching the slow pharmacokinetics of antibodies. The increasing number of clinical studies performed with ^89^Zr-labeled antibodies also reflects the clinical relevance of the compound class [1].

**Figure 1:**
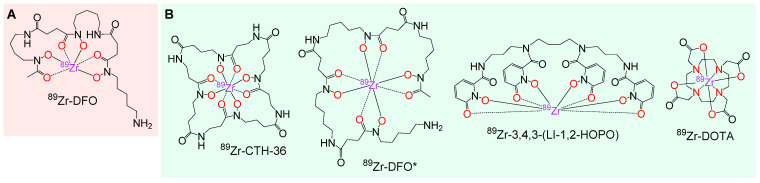
Structures of the complexes [^89^Zr]Zr-DFO (**A**) and [^89^Zr]Zr-DFO*, [^89^Zr]Zr-CTH36, [^89^Zr]Zr-3,4,3-(LI-1,2-HOPO) and [^89^Zr]Zr-DOTA (**B**).

In clinical studies, ^89^Zr^4+^ is usually introduced into the biomolecule carrier using desferrioxamine B (DFO; Figure 1A), a natural siderophore. However, there is strong evidence that the kinetic inertness of the formed ^89^Zr–DFO complex is limited [2,3,4], resulting in release of the ^89^Zr cation. Free ^89^Zr^4+^ attaches to the hydroxyapatite of bones, resulting in significant uptake into bones and joints. This is of course problematic, as it can result in a relevant dose to hematopoietic bone marrow and, furthermore, reduces the quality of the obtained images. On the one hand, this is caused by the higher background accumulation of freely circulating ^89^Zr^4+^, and on the other hand, the bone uptake compromises the visualization of bone metastases.

For this reason, several new chelating agents have been developed over the last years, some of which have been shown to be significantly more suitable than DFO to form kinetically inert complexes with ^89^Zr [3,5]. All of these attempts to develop stable ^89^Zr complexes rely on the complete saturation of the coordination sphere of ^89^Zr^4+^. In the ^89^Zr–DFO complex, the three hydroxamates occupy only six of the preferred eight coordination sites of the Zr^4+^ ion, leaving a gap in the ligand sphere where other ions and molecules can interact, destabilize or break the complex. A complete saturation of the coordination sphere, together with a complete spatial embedment of the central ion, are thus equally important for the formation of stable 8^9^Zr complexes.

There has been no comparison of these most promising representatives of this class of new chelating agents for ^89^Zr regarding their kinetic inertness. It is thus still not clear which chelator is the most suitable for clinical translation to replace the commonly used gold standard DFO in clinical applications.

Very recently, a highly interesting study reported on the prediction of the thermodynamic stability of different ^89^Zr-based radiotracers [6]. Here, the absolute and relative formation constants of 23 different zirconium complexes were determined by means of density functional theory (DFT) calculations. In this study, which differentiated between DFO chelator analogs and alternative chelating agents, it was shown that some of the complexes investigated exhibited very promising thermodynamic stabilities.

In the group of alternative chelating agents, the [^89^Zr]Zr–CTH36 complex (Figure 1B) deserves special consideration, as it exhibited a particularly high complexation constant of *β* = 52.84. This is the result of a nearly optimal complex geometry that is close to the lowest energy structure of Zr(MeAHA)_4_, being formed by Zr^4+^ and bidentate MeAHA (*N*-methyl-acetohydroxamic acid), and the macrocyclic structure of the chelator. This macrocyclic structure results in a preorganization of the hydroxamates and in a reduced entropic penalty during complex formation compared to acyclic chelates (such as DFO). The high flexibility, due to the eight atom chains between each set of carbonyl and N−O donor groups, further reduces steric strain and allows the donor atoms to adopt the preferred geometry.

Within the group of DFO-based chelators, [^89^Zr]Zr-DFO* (Figure 1B) also showed a very high formation constant of *β* = 51.56, which can be explained by the complete saturation of the coordination sphere of the Zr^4+^ ion.

These theoretical considerations are supported by experimental studies demonstrating that the chelators CTH36 and DFO* form complexes of significantly increased stability compared to DFO in in silico complex challenges and/or in vivo imaging studies [7,8,9,10]. Therefore, these two chelating agents are of high interest with regard to further comparative investigation and also potential clinical application.

Other chelating agents that showed a significantly higher stability of the formed ^89^Zr complexes were 3,4,3-(LI-1,2-HOPO) [11,12] and DOTA (1,4,7,10-tetraazacyclododecane-1,4,7,10-tetrayl)tetraacetic acid) [13] (Figure 1B), which are thus additional promising candidates for further comparative investigation of complex stability and clinical applicability.

The aim of the current study was therefore to directly compare the mentioned four chelating agents, as well as the commonly used DFO with regard to the relative kinetic inertness of the ^89^Zr complexes formed under identical conditions for direct comparability of the obtained results, and to be able to identify the most useful chelating agent for stable ^89^Zr complexation.

For this, analogs of these chelating agents were to be developed enabling an efficient introduction into biomolecules by a chemoselective and biorthogonal conjugation reaction to facilitate a high-yield derivatization of even sensitive biomolecules such as antibodies. For this purpose, a necessary functional group for bioconjugation had to be introduced in a position of the molecular structure of the chelators not interfering with ^89^Zr complex formation. This requires a backbone functionalization of the respective chelators, leaving the hydroxamate or carboxylate functional groups needed for ^89^Zr complexation uncompromised. Furthermore, the same biorthogonal and chemoselective conjugation reaction should find application in all cases, thus excluding the possibility that the bioconjugation chemistry itself influences ^89^Zr complex formation or kinetic inertness.

A popular and customizable click chemistry reaction is the inverse electron demand Diels-Alder (iEDDA) conjugation reaction between tetrazines and TCOs (TCO = trans-cyclooctene), which has already found widespread application in radiochemistry [14,15,16]. For this reason, we decided (i) to synthesize backbone tetrazine-modified analogs of DFO, CTH-36, DFO*, 3,4,3-(LI-1,2-HOPO) and DOTA, leaving the coordination sphere of the respective agents unaltered to achieve a high kinetic inertness of the resulting ^89^Zr complexes, and (ii) to introduce them into c(RGDfK); (iii) to radiolabel the resulting bioconjugates with ^89^Zr and (iv) to determine the inertness of the resulting equally modified and conjugated ^89^Zr complexes by challenge experiments under identical conditions to be able to directly compare and evaluate their relative stability, with the aim to identify the most promising candidate for stable ^89^Zr complexation.

## 2. Materials and Methods

### 2.1. General

All solvents and reagents were used without further purification. Acetonitrile and water (HPLC grade) were obtained from Häberle Labortechnik (Lonsee-Ettlenschieß, Germany). TFA (uvasol quality) for HPLC and all anhydrous solvents were purchased from Sigma-Aldrich (Taufkirchen, Germany). H_2_O (Tracepur quality), hydrochloric acid (30%, Suprapur quality), sodium hydroxide (30%, Suprapur quality) and the protected amino acid derivatives used for solid phase-based peptide synthesis, PyBOP (Benzotriazole-1-yl-oxy-tris-pyrrolidino-phosphonium hexafluorophosphate), as well as the Fmoc-Asp(NovaSynTGA)-OAll resin, were purchased from Merck (Darmstadt, Germany). HBTU (2-(1*H*-Benzotriazole-1-yl)-1,1,3,3-tetramethyluronium hexafluorophosphate) and DIPEA (*N*,*N*-diisopropylethylamine) and TFA (trifluoroacetic acid) were obtained from Carl Roth (Karlsruhe, Germany) and Sigma Aldrich (Taufkirchen, Germany), respectively. (4-(1,2,4,5-tetrazine-3-yl)phenyl)methanamine hydrochloride and 4-(1,2,4,5-tetrazine-3-yl)benzoic acid were purchased from Varimol (Stuttgart, Germany). HEPES (4-(2-hydroxyethyl)piperazine-1-ethanesulfonic acid) (ultrapure quality) was obtained from Gerbu Biotechnik GmbH (Heidelberg, Germany). Dulbecco’s phosphate-buffered saline (DPBS) was obtained from VWR (Bruchsal, Germany). [^89^Zr]Zr-oxalate solution in 1.0-M oxalic acid was purchased from PerkinElmer (NEZ308000MC, Rodgau, Germany). All other standard chemicals and solvents were obtained from Carl Roth (Karlsruhe, Germany), Sigma Aldrich (Taufkirchen, Germany), TCI Deutschland GmbH (Eschborn, Germany) and Thermo Fisher GmbH (Kandel, Germany). Sep-Pak Light (46 mg) Accell Plus QMA Carbonate cartridges were obtained from Waters (Eschborn, Germany).

**2 [10]**, **5 [17]**, **9**–**13 [18]**, **15 [18]**, **22** [19], **23**–**24 [20]**, **28 [10]** and **30 [10]** were synthesized according to literature protocols. All compounds were obtained in at least 95% purity unless otherwise stated.

Analytical and semipreparative high-performance liquid chromatography (HPLC) analyses and purifications were carried out on Dionex UltiMate 3000 systems (Thermo Fisher) equipped with Chromolith Performance (RP-18e, 100–4.6 mm, Merck, Darmstadt, Germany) or Chromolith SemiPrep (RP-18e, 100–10 mm, Merck) columns, respectively. A flow rate of 4 mL/min and the eluents H_2_O and acetonitrile (MeCN) containing either 0.1% trifluoroacetic acid (TFA) or 0.1% formic acid (FA) were used. Nuclear magnetic resonance (NMR) spectroscopy was carried out on a 500-MHz Varian NMR System spectrometer, a 700-MHz Bruker Avance III HD NMR spectrometer and a 300-MHz MERCURYplus NMR spectrometer, respectively. The signals of the deuterated solvents were used as references. All chemical shifts (*δ*) are reported in ppm and the coupling constants (*J*) in Hz. The matrix-assisted laser desorption/ionization mass spectroscopy (MALDI-MS) was carried out with on a Bruker Daltonics Microflex spectrometer (Bremen, Germany) and for the high-resolution electrospray ionization mass spectroscopy (HR-ESI-MS), a Thermo Finnigan LTQ FT Ultra Fourier Transform Ion Cyclotron Resonance (Dreieich, Germany) mass spectrometer was used. Radioactivity was measured using an ISOMED 2010 (Kappeln, Germany) activimeter. Analytical radio-HPLC chromatography was performed on a Dionex UltiMate 3000 system (Thermo Fischer, Dreieich, Germany) equipped with a radio detector GabiStar (Raytest) and a Gemini column (C18, 5 µm, 250–4.6 mm, Phenomenex) at a flow rate of 2 mL/min using the eluents H_2_O and MeCN containing 0.1% TFA. As the gamma counter, the 2480 Wizard system (PerkinElmer) was used. Radio-iTLC (instant thin-layer chromatography) analyses were carried out using ITLC-SG strips (Agilent Technologies) together with citrate buffer as the eluent (0.1M, pH 5), which were analyzed using a Scan-RAM radio-TLC scanner (LabLogic) using LAURA software (Jahnsdorf, Germany, for the analyses of radio-HPLC, TLC and GC chromatography, version: 4.1.12.89).

### 2.2. Syntheses of Chelator Tetrazines

#### 2.2.1. 4-((4-(1,2,4,5-Tetrazin-3-yl)benzyl)amino)-4-oxobutanoic Acid **7**

**Scheme 1:**
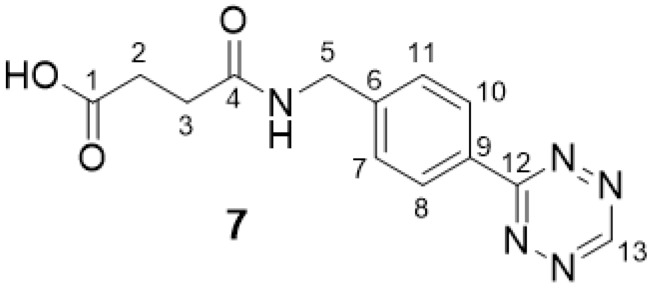
Structure of **7**.

(4-(1,2,4,5-Tetrazin-3-yl)phenyl)methanamine hydrochloride **6** (15 mg, 67.1 µmol) was added to a solution of succinic anhydride (8 mg, 80.5 µmol) in DMF (1 mL). After the addition of triethylamine (9.3 µL, 67.1 µmol), the mixture was stirred under exclusion of light for 4 h at ambient temperature. Then, the solvent was removed under reduced pressure, and the crude product was purified by semipreparative HPLC using a gradient of 0–40% MeCN + 0.1% TFA in 8 min (*R*_t_ = 5.51 min). Finally, the product was isolated as pink solid in a yield of 86% (17 mg, 57.8 µmol). ^1^H NMR (500 MHz, DMSO-*d*_6_, 25 °C) *δ* 12.11 (s, 1H, O*H*), 10.57 (s, 1H, *H*-13), 8.52 (t, *J* = 5.9 Hz, 1H, N*H*), 8.44 (d, *J* = 8.3 Hz, 2H, *H*-8, *H*-10), 7.54 (d, *J* = 8.3 Hz, 2H, *H*-7, *H*-11), 4.41 (d, *J* = 6.0 Hz, 2H, *H*-5), 2.53–2.47 (m, 2H, *H*-2), 2.47–2.41 (m, 2H, *H*-3). ^13^C-NMR (125 MHz, DMSO-*d*_6_, 25 °C) *δ* 173.88 (*C*-1), 171.30 (*C*-4), 165.44 (*C*-12), 158.11 (*C*-13), 145.00 (*C*-9), 130.29 (*C*-6), 128.02 (*C*-7, *C*-11), 127.75 (*C*-8, *C*-10), 41.87 (*C*-5), 30.02 (*C*-3), 29.13 (*C*-2). MALDI-MS (*m*/*z*) for [M + H^+^] (calculated): 288.26 (288.11). See Scheme 1 for molecular formula.

#### 2.2.2. DFO Tetrazine **1**

**Scheme 2:**
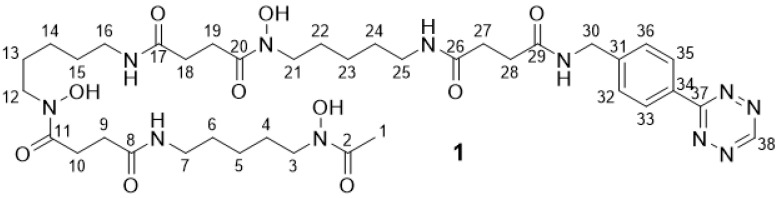
Structure of **1**.

4-((4-(1,2,4,5-Tetrazin-3-yl)benzyl)-amino)-4-oxobutanoic acid **7** (7.0 mg, 24.4 µmol) and DFO mesylate (17.6 mg, 26.8 µmol) were dissolved in DMSO (0.75 mL). PyBOP (25 mg, 48.7 µmol) and triethylamine (102 µL, 73.1 µmol) were added, and the reaction was stirred for 3 h under exclusion of light at ambient temperature. The crude product was precipitated with the addition of MeCN/water (1/1, *v*/*v*). The obtained solid was redissolved in DMSO and reprecipitated. This process was repeated twice. The product was isolated as pink solid after lyophilization in a yield of 65% (13.2 mg, 15.9 µmol). ^1^H-NMR (500 MHz, DMSO-*d*_6_, 25 °C) *δ* 10.58 (s, 1H, *H*-38), 9.64 (s, 1H, O*H*), 9.62–9.54 (m, 2H, O*H*), 8.49 (t, *J* = 6.1 Hz, 1H, N*H*), 8.45 (d, *J* = 8.4 Hz, 2H, *H*-33, *H*-35), 7.81 (t, *J* = 5.6 Hz, 1H, N*H*), 7.77 (t, *J* = 5.6 Hz, 2H, N*H*), 7.53 (d, *J* = 8.3 Hz, 2H, *H*-32, *H*-36), 4.39 (d, *J* = 6.0 Hz, 2H, *H*-30), 3.49–3.41 (m, 6H, *H*-3, *H*-12, *H*-21), 3.07–2.94 (m, 6H, *H*-7, *H*-16, *H*-25), 2.57 (t, *J* = 7.4 Hz, 4H, *H*-10, *H*-19), 2.42 (t, *J* = 6.9 Hz, 2H, *H*-28), 2.35 (t, *J* = 6.9 Hz, 2H, *H*-27), 2.26 (t, *J* = 7.4 Hz, 4H, *H*-9, *H*-18), 1.96 (s, 3H, *H*-1), 1.55–1.44 (m, 6H, *H*-4, *H*-13, *H*-22), 1.43–1.32 (m, 6H, *H*-6, *H*-15, *H*-24), 1.27–1.16 (m, 6H, *H*-5, *H*-14, *H*-23). ^13^C-NMR (125 MHz, DMSO-*d*_6_, 25 °C) *δ* 172.41 (*C*=O), 172.13(*C*=O), 171.74 (*C*=O), 171.52 (*C*=O), 170.58 (*C*-2), 165.88 (*C*-37), 158.55 (*C*-38), 145.47 (*C*-34), 130.74 (*C*-31), 128.46 (*C*-32, *C*-36), 128.19 (*C*-33, *C*-35), 47.52 und 47.23 (*C*-3, *C*-12, *C*-21), 42.29 (*C*-30), 40.88, 38.87 und 38.86 (*C*-7, *C*-16, *C*-25), 31.29 (*C*-28), 31.22 (*C*-27), 30.34 (*C*-9, *C*-18), 29.27 (*C*-6, *C*-15, *C*-24), 28.01 (*C*-10, *C*-19), 26.48 (*C*-4, *C*-13, *C*-22), 23.94 (*C*-5, *C*-14, *C*-23), 20.79 (*C*-1). MALDI-MS (*m*/*z*) for [M + H]^+^ (calculated): 829.66 (830.45), [M + Na]^+^ (calculated): 852.82 (852.43), [M + K]^+^ (calculated): 868.34 (868.41). HR-ESI-MS (*m*/*z*) for [M + H]^+^ (calculated): 830.4508 (830.4519), [M + Na]^+^ (calculated): 852.4327 (852.4339), [M − H]^−^ (calculated): 828.4374 (828.4374). See Scheme 2 for molecular formula.

#### 2.2.3. O-Benzyl-N-(5-(((benzyloxy)carbonyl)amino)pentyl)hydroxylammonium Chloride **14**

**Scheme 3:**
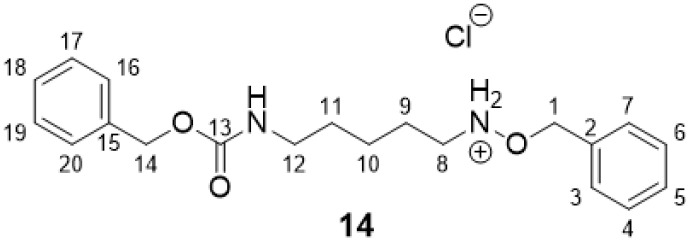
Structure of **14**.

^t^Butyl(benzyloxy)(5-(1,3-dioxoisoindolin-2-yl)pentyl)-carbamate **13** (2.0 g, 4.52 mmol) was dissolved in 1,4-dioxane (18 mL). A solution of HCl in 1,4-dioxane (18 mL, 4 M) was added while cooling the reaction in an ice bath. After stirring the reaction for 4 h at ambient temperature, the solvent was evaporated under reduced pressure. The crude product was washed with diethylether using the ultrasonic bath. The product was isolated as a colorless solid in a yield of 79% (1.355 g, 3.58 mmol). ^1^H-NMR (500 MHz, CDCl_3_, 25 °C) *δ* 12.29 (s, 2H, N*H*), 7.44–7.27 (m, 10H, *H*-3, *H*-4, *H*-5, *H*-6, *H*-7, *H*-16, *H*-17, *H*-18, *H*-19, *H*-20), 5.34 (s, 2H, *H*-1), 5.07 (s, 2H, *H*-14), 3.29–3.21 (m, 2H, *H*-8), 3.15 (t, *J* = 6.3 Hz, 2H, *H*-12), 1.95–1.84 (m, 2H, *H*-9), 1.50 (m, 2H, *H*-11), 1.46–1.37 (m, 2H, *H*-10). ^13^C-NMR (125 MHz, DMSO-*d*_6_, 25 °C) *δ* 156.73 (*C*-13), 136.66 (*C*-15), 132.70 (*C*-2), 129.72 (*C*H_Ar_), 129.68 (*C*H_Ar_), 128.93 (*C*H_Ar_), 128.64 (*C*H_Ar_), 128.22 (*C*H_Ar_), 128.15 (*C*H_Ar_), 76.70 (*C*-1), 66.84 (*C*-14), 49.52 (*C*-8), 40.71 (*C*-12), 29.32 (*C*-11), 23.79 (*C*-10), 23.34 (*C*-9). MALDI-MS (*m*/*z*) for [M + H]^+^ (calculated): 342.78 (343.21), [M + Na]^+^ (calculated): 364.66 (365.18), [M + K]^+^ (calculated): 380.55 (381.16). HR-ESI-MS (*m*/*z*) for [M + H]^+^ (calculated): 343.2018 (243.2017), [M + Na]^+^ (calculated): 365.1842 (365.1836). See Scheme 3 for molecular formula.

#### 2.2.4. Bn-Cbz-DFO* **16**

**Scheme 4:**
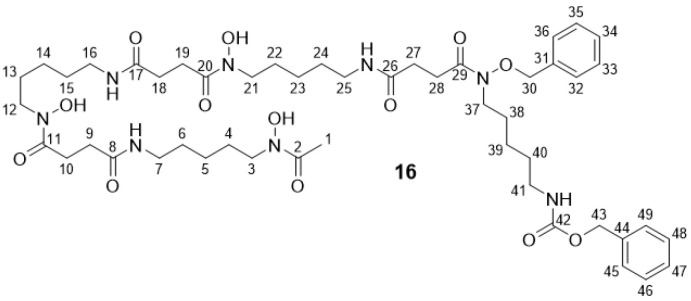
Structure of **16**.

4-((Benzyloxy)(5-(((benzyloxy)-carbonyl)-amino)pentyl)amino)-4-oxo-butanoic acid **15** (177 mg, 0.26 mmol) was dissolved in DMF (1.0 mL), followed by the addition of PyBOP (197 mg, 0.38 mmol) and DFO mesylate (170 mg, 0.26 mmol). DIPEA (136 µL, 0.78 mmol) was added, and the reaction mixture was stirred for 30 h. DMF was evaporated under reduced pressure, and the residue was washed three times with acetone (3 mL) and three times with distilled water (3 mL). After lyophilization, the product was obtained as a colorless solid in a yield of 67%. ^1^H-NMR (500 MHz, DMSO-*d*_6_, 25 °C) *δ* 9.72–9.57 (O*H*, m, 3H, O*H*), 7.78 (d, *J* = 2.1 Hz, 3H, N*H*), 7.48–7.27 (m, 10H, *H*-Ar), 7.22 (t, *J* = 5.5 Hz, 1H, N*H*), 4.99 (s, 2H, *H*-30), 4.88 (s, 2H, *H*-43), 3.56 (t, *J* = 6.6 Hz, 2H, *H*-37), 3.45 (t, *J* = 6.8 Hz, 6H, *H*-3, *H*-12, *H*-21), 3.35 (s, 2H), 3.06–2.93 (m, 8H, *H*-7, *H*-16, *H*-25, *H*-41), 2.60 (dt, *J* = 13.9, 6.3 Hz, 6H, *H*-10, *H*-19, *H*-28), 2.29 (dt, *J* = 18.7, 7.1 Hz, 6H, *H*-9, *H*-18, *H*-27), 1.96 (s, 3H, *H*-1), 1.57–1.44 (m, 8H, *H*-4, *H*-13, *H*-22, *H*-38), 1.43–1.33 (m, 8H, *H*-6, *H*-15, *H*-24, *H*-40), 1.27–1.15 (m, 8H, *H*-5, *H*-14, *H*-23, *H*-39). ^13^C-NMR (125 MHz, DMSO-*d*_6_, 25 °C) *δ* 171.96 (*C*=O), 171.29 (*C*=O), 170.99 (*C*=O), 156.07 (*C*=O), 137.30 (*C*-Ar), 134.96 (*C*-Ar), 129.25 (*C*-Ar), 128.61 (*C*-Ar), 128.48 (*C*-Ar), 128.32 (*C*-Ar), 127.69 (*C*-Ar), 75.42 (*C*-43), 65.08 (*C*-30), 47.08 and 46.79 (*C*-3, *C*-12, *C*-21), 44.42 (*C*-37), 40.13, 40.02, 39.86, 38.43 (*C*-7, *C*-16, *C*-25, *C*-41), 29.91 and 29.70 (*C*-9, *C*-18, *C*-27), 29.01 and 28.82 (*C*-6, *C*-15, *C-*24), 27.58 and 27.21 (*C*-10, *C*-19, *C*-28), 26.04 (*C*-4, *C*-13, *C-*22, *C*-38), 23.50 and 23.38 (*C*-5, *C*-14, *C*-23, *C*-39), 20.35 (*C*-1). MALDI-MS (*m*/*z*) for [M + H]^+^ (calculated): 985.40 (985.56), [M + Na]^+^ (calculated): 1007.50 (1007.54), [M + K]^+^ (calculated): 1023.48 (1024.27). HR-ESI-MS (*m*/*z*) for [M + H]^+^ (calculated): 985.5603 (985.5605), [M + Na]^+^ (calculated): 1007.5425 (1007.5425), [M − H]^−^ (calculated): 983.5459 (983.5459). See Scheme 4 for molecular formula.

#### 2.2.5. DFO* **17**

**Scheme 5:**
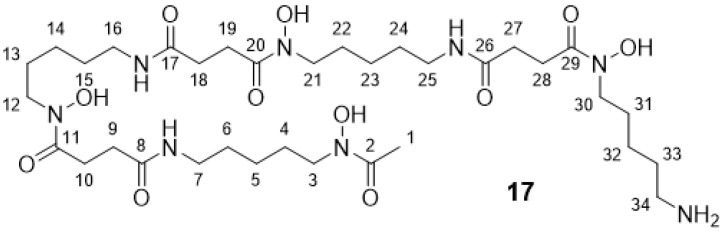
Structure of **17**.

The synthesis was carried out according to the literature, with minor changes [7]. Bn-DFO*-Cbz (100 mg, 0.10 mmol) was dissolved in methanol (80 mL). A small amount (10%) of Pd/C (30 mg) was added. The reaction was stirred under hydrogen gas atmosphere (balloon) for 12 h at ambient temperature. The Pd/C was filtered off, and DFO* was obtained after the evaporation of methanol in a yield of 70% (54 mg, 0.07 mmol). ^1^H-NMR (500 MHz, DMSO-*d*_6_, 25 °C) *δ* 7.79 (s, 3H, N*H*), 3.45 (t, *J* = 7.0 Hz, 8H, *H*-3, *H*-12, *H*-21, *H*-30), 2.99 (dd, *J* = 12.5, 6.3 Hz, 6H, *H*-7, *H*-16, *H*-25), 2.61 (dt, *J* = 13.5, 7.0 Hz, 6H, *H*-10, *H*-19, *H*-28), 2.26 (t, *J* = 7.0 Hz, 6H, *H*-9, *H*-18, *H*-27), 1.96 (s, 3H, *H*-1), 1.55–1.45 (m, 8H, *H*-4, *H*-13, *H*-22, *H*-31), 1.41–1.32 (m, 8H, *H*-6, *H*-15, *H*-24, *H*-33), 1.25–1.18 (m, 8H, *H*-5, *H*-14, *H*-23, *H*-32). ^13^C-NMR (125 MHz, DMSO-*d*_6_, 25 °C) *δ* 171.92 (*C*=O), 171.31 (*C*=O), 171.16 (*C*=O), 170.10 (*C*=O), 47.08 und 46.78 (*C*-3, *C*-12, *C*-21, *C*-30), 38.41 (*C*-7, *C*-16, *C*-25), 30.94, 29.94 (*C*-9, *C*-18, *C*-27), 29.25, 28.81 (*C*-6, *C*-15, *C*-24, *C*-33), 27.59 (*C*-10, *C*-19, *C*-28), 26.04 (*C*-4, *C*-13, *C*-22, *C*-31), 23.50 (*H*-14, *H*-23, *H*-32), 23.10, 20.36 (*C*-1). MALDI-MS (*m*/*z*) for [M + H]^+^ (calculated): 761.32 (761.48), [M + Na]^+^ (calculated): 783.46 (783.34). HR-ESI-MS (*m*/*z*) for [M + H]^+^ (calculated): 761.4774 (761.4767), [M − H]^−^ (calculated): 759.4628 (759.4622). See Scheme 5 for molecular formula.

#### 2.2.6. DFO* Tetrazine **3**

**Scheme 6:**
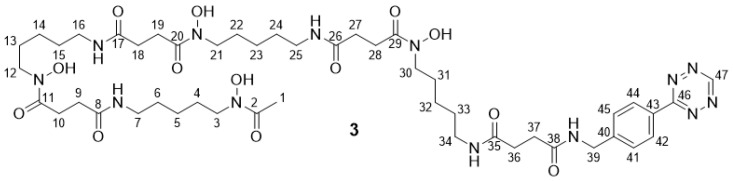
Structure of **3**.

4-((4-(1,2,4,5-Tetrazin-3-yl)benzyl)amino)-4-oxo-butanoic acid **7** (6.5 mg, 22.6 µmol) and DFO* **17** (17.2 mg, 22.6 µmol) were dissolved in DMSO (0.7 mL). PyBOP (23.5 mg, 45.3 µmol) and triethylamine (9.5 µL, 67.9 µmol) were added, and the reaction was stirred for 3 h under exclusion of light at ambient temperature. The crude product was precipitated with the addition of MeCN/water (1:1). The solid was redissolved in DMSO and reprecipitated. This procedure was repeated twice. The purification of the product was performed on semipreparative HPLC using a gradient of 0–50% MeCN + 0.1% FA in 10 min at 40 °C (*R*_t_ = 6.35 min). After lyophilization, the product was isolated in a yield of 19% (4.4 mg, 4.3 µmol). ^1^H-NMR (500 MHz, DMSO-*d*_6_, 25 °C) *δ* 10.57 (s, 1H, *H*-47), 9.67 (s, 4H, N-O*H*), 8.49 (t, *J* = 6.0 Hz, 1H, N*H*), 8.45 (d, *J* = 8.3 Hz, 2H, *H*-42, *H*-44), 7.84–7.79 (m, 1H, N*H*), 7.77 (t, *J* = 5.1 Hz, 3H, N*H*), 7.54 (d, *J* = 8.1 Hz, 2H, *H*-41, *H*-45), 4.39 (d, *J* = 5.7 Hz, 2H, *H*-39), 3.45 (t, *J* = 6.4 Hz, 8H, *H*-3, *H*-12, *H*-21, *H*-30), 3.06–2.92 (m, 8H, *H*-7, *H*-16, *H*-25, *H*-34), 2.57 (t, *J* = 6.8 Hz, 6H, *H*-10, *H*-19, *H*-28), 2.45–2.39 (m, 2H, *H*-36 or *H*-37), 2.38–2.32 (m, 2H, *H*-36 or *H*-37), 2.26 (t, *J* = 7.2 Hz, 6H, *H*-9, *H*-18, *H*-27), 1.96 (s, 3H, *H*-1), 1.55–1.43 (m, 8H, *H*-4, *H*-13, *H*-22, *H*-31), 1.42–1.31 (m, 8H, *H*-6, *H*-15, *H*-24, *H*-33), 1.29–1.14 (m, 10H, *H*-5, *H*-14, *H*-23, *H*-32). ^13^C-NMR (125 MHz, DMSO-*d*_6_, 25 °C) *δ* 171.92 (*C*=O), 171.66 (*C*=O), 171.28 (*C*=O), 171.05 (*C*=O), 165.41 (*C*-46), 145.00 (*C*-43), 130.27 (*C*-40), 128.00 (2 × *C*-Ar), 127.72 (2 × *C*-Ar), 47.07 and 46.77 (*C*-3, *C*-12, *C*-21, *C*-30), 41.83 (*C*-39), 38.41 (*C*-7, *C*-16, *C*-25, *C*-34), 30.85 (*C*-36 or *C*-37), 30.77 (*C*-36 or *C*-37), 29.91(*C*-9, *C*-18, *C*-27), 28.80 (*C*-6, *C*-15, *C*-24), 27.57 (*C*-10, *C*-19, *C*-28), 26.02 (*C*-4, *C*-13, *C*-22), 23.49 (*C*-5, *C*-14, *C*-23, *C*-32), 20.33 (*C*-1). MALDI-MS (*m*/*z*) for [M + H]^+^ (calculated): 1030.20 (1030.57), [M + Na]^+^ (calculated): 1052.62 (1052.55), [M + K]^+^ (calculated): 1069.01 (1069.52). HR-ESI-MS (*m*/*z*) for [M + H]^+^ (calculated): 1030.5667 (1030.5680), [M + Na]^+^ (calculated): 1052.5490 (1052.5500), [M − H]^−^ (calculated): 1028.5528 (1028.5535). See Scheme 6 for molecular formula.

#### 2.2.7. Tris-Boc-spermine-trifluoroacetamide **18** and Tris-Boc-spermine **19**

**Scheme 7:**
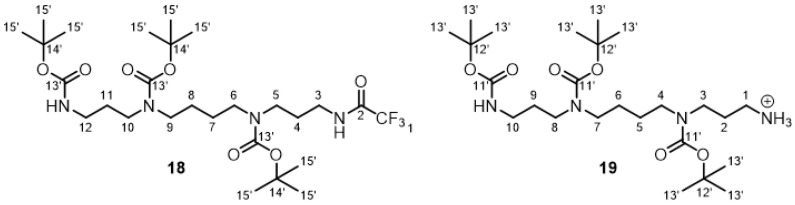
Structure of **18** and **19**.

The synthesis of both compounds was carried out following in principle a published procedure [12]. Spermine (526 mg, 2.60 mmol) was dissolved in methanol (40 mL), and the solution was cooled in an acetone–liquid nitrogen cooling bath to −78 °C. A solution of ethyl trifluoroacetate (0.44 mL, 2.6 mmol) in methanol (26 mL) was added dropwise over one hour under argon atmosphere. The reaction mixture was stirred another 30 min at −78 °C before the temperature was slowly increased to 0 °C. A solution of di-*tert*-butyldicarbonate (3.583 g, 15.60 mmol) in methanol (26 mL) was added, and the reaction was stirred overnight. The solvent was removed on a rotary evaporator, and the obtained residue was redissolved in DCM (20 mL) and washed three times with water (15 mL). The crude product **18** was purified by column chromatography using a gradient of 30–100% ethyl acetate in cyclohexane + 1% NH_3_ (aq.). The product could be isolated in pure form, but residues of *tetra*-Boc-spermine-trifluoroacetamide were still contained. ^1^H-NMR (300 MHz, CDCl_3_, 25 °C) *δ* 8.25 (s, 1H, N*H*), 3.36–3.05 (m, 12H, *H*-3, *H*-5, *H*-6, *H*-9, *H*-10, *H*-12), 1.75–1.60 (m, 4H, *H*-4, *H*-4), 1.53–1.47 (m, 4H, *H*-7, *H*-8), 1.45 (s, 18H, *H*_Boc_), 1.43 (s, 9H, *H*_Boc_). MALDI-MS (*m*/*z*) for [M + Na]^+^ (calculated): 620.92 (621.34), [*tetra*-Boc-spermine- trifluoroacetamide + Na]^+^ (calculated): 625.01 (625.41). **18** was used in the next step without further purification. For this purpose, **18** was dissolved in methanol (40 mL), and a 30% ammonia solution was added to adjust the pH to 11–12. The mixture was stirred for four days, until no more **18** was detected. The product **19** was obtained as a mixture with *tetra*-Boc-spermine (overall yield: quant.). ^1^H-NMR (500 MHz, CDCl_3_, 25 °C) *δ* 8.51–8.12 (m, 5H, N*H*), 3.48–2.96 (m, 12H, *H*-1, *H*-3, *H*-4, *H*-7, *H*-8, *H*-10), 2.32 (s, 1H), 2.06 (s, 1H), 1.86–1.52 (m, 4H, *H*-2, *H*-9), 1.52–1.46 (m, 4H, *H*-5, *H*-6), 1.45 (s, 18H, *H*-Boc), 1.43 (s, 9H, *H*-Boc). ^13^C-NMR (125 MHz, CDCl_3_, 25 °C) *δ* 157.88, 157.59, 157.30, 157.01, 156.15, 155.63, 124.95, 117.30, 115.00, 110.14, 80.46, 79.77, 79.09, 53.55, 46.98, 45.28, 44.39, 43.96, 43.12, 37.88, 37.52, 35.97, 29.83, 28.61, 28.59, 28.58, 28.50, 28.37, 28.17, 27.89, 27.34, 26.12. MALDI-MS (*m*/*z*) for [M + H]^+^ (calculated): 502.43 (503.38). See Scheme 7 for molecular formula.

#### 2.2.8. 1-Hydroxy-6-oxo-1,6-dihydropyridine-2-carbonic Acid **21**

**Scheme 8:**
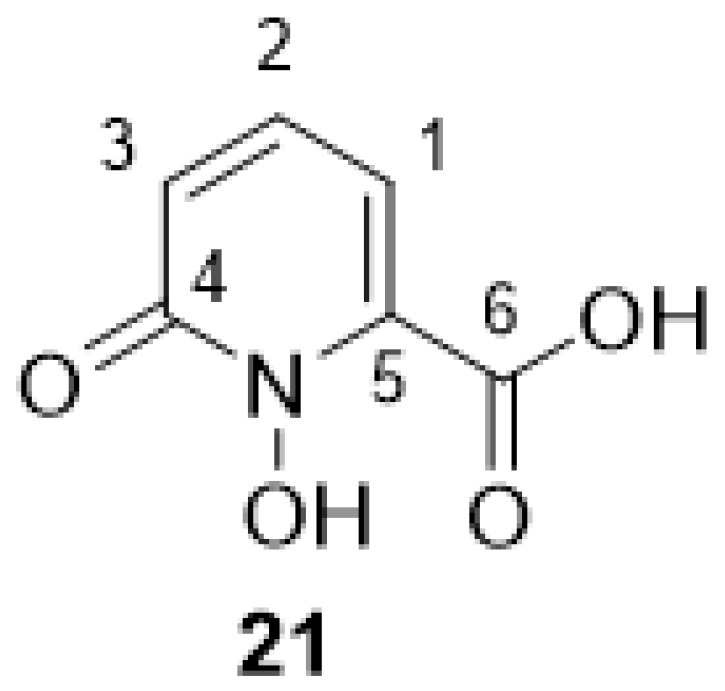
Structure of **21**.

The synthesis was carried out on the basis of the literature protocol with minor changes [20]. 6-Hydroxypicolinic acid (2.00 g, 14.38 mmol) was dissolved in acetic acid (8.2 mL) and neat TFA (12.2 mL). Under argon atmosphere, a 35% solution of peracetic acid in acetic acid (6.9 mL, 36.82 mmol) was added, and the reaction was stirred 1 h at ambient temperature, followed by stirring 17 h at 80°C. Next, the mixture was cooled at 4 °C for 12 h. The formed precipitate was filtered off and washed with ice-cold methanol. Compound **21** was obtained as a colorless solid in a yield of 69% (1.545 g, 4.149 mmol). ^1^H-NMR (500 MHz, DMSO-*d*_6_, 25 °C) *δ* 10.05 (s, 1H, O*H*), 7.45 (dd, *J* = 9.0, 7.0 Hz, 1H, *H*-2), 6.72 (dd, *J* = 9.0, 1.6 Hz, 1H, *H*-3), 6.64 (dd, *J* = 7.0, 1.5 Hz, 1H, *H*-1). ^13^C-NMR (125 MHz, DMSO-*d*_6_, 25 °C) *δ* 161.85 (*C*-6), 157.15 (*C*-4), 138.97 (*C*-5), 136.76 (*C*-2), 120.32 (*C*-3), 106.33 (*C*-1). HR-ESI-MS (*m*/*z*) for [M − H]^−^ (calculated): 154.0146 (154.0145). See Scheme 8 for molecular formula.

#### 2.2.9. ^t^Butyl-(4-(2-((3-((4-((3-aminopropyl)amino)butyl)amino)propyl)amino)ethyl)phenyl)-carbamate **25**

**Scheme 9:**
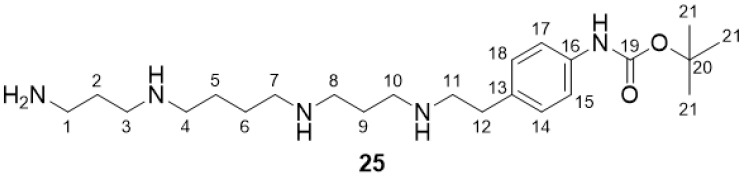
Structure of **25**.

The synthesis was carried out on the basis of the literature protocol [20]. Spermine (300 mg, 1.48 mmol) was dissolved in acetonitrile (45 mL), and potassium carbonate (410 mg, 2.97 mmol) was added. A solution of 4-((*tert*-butoxy-carbonyl)amino)-phenethyl-4-methylbenzenesulfonate **24** (296 mg, 0.76 mmol) in acetonitrile (45 mL) was prepared and added dropwise while cooling with ice. After the reaction was refluxed for 16 h, the potassium carbonate was filtered off, and the solvent was removed on a rotary evaporator. The residue was redissolved in acetonitrile/water and purified via semipreparative HPLC using a gradient 0–35% MeCN + 0.1% FA in 8 min (*R*_t_ = 6.35 min). After lyophilization, the product was isolated as a colorless solid in a yield of 63% (202 mg, 48 mmol). ^1^H-NMR (500 MHz, CDCl_3_, 25 °C) *δ* 9.32 (s, 1H, N*H*), 9.05–8.85 (m, 5H, N*H*), 8.25–7.93 (m, 4H, N*H*), 7.51 (d, *J* = 8.0 Hz, 0.2H, *H*-15, *H*-17), 7.40 (d, *J* = 8.2 Hz, 0.5H, *H*-15, *H*-17), 7.17 (d, *J* = 8.5 Hz, 2H, *H*-14, *H*-18), 7.12 (d, *J* = 8.5 Hz, 2H, *H*-14, *H*-18), 4.16 (s, 3H, TFA), 3.27–2.80 (m, 16H, *H*-1, *H*-3, *H*-4, *H*-7, *H*-8, *H*-10, *H*-11, *H*-12), 2.05–1.86 (m, 4H, *H*-2, *H*-9), 1.81–1.60 (m, 4H, *H*-5, *H*-6), 1.45 (s, 9H, *H*-21). ^13^C-NMR (125 MHz, CDCl_3_, 25 °C) *δ* 158.83, 158.57, 152.80, 138.29, 130.38, 128.99, 128.79, 128.23, 125.49, 118.14, 115.77, 113.40, 78.98 (*C*-20), 53.09, 51.54, 48.91, 47.87, 46.11, 43.87, 36.19, 30.89, 28.13 (*C*-21), 23.78, 22.68, 22.64, 22.62, 22.32. MALDI-MS (*m*/*z*) for [M + H]^+^ (calculated): 422.02 (422.35). HR-ESI-MS (*m*/*z*) for [M + H]^+^ (calculated): 422.3491 (422.3490), [M + HCO]^+^ (calculated): 450.3439 (450.3439). See Scheme 9 for molecular formula.

#### 2.2.10. 3,4,3-(LI-1,2-HOPOBn)-NH-Boc **26**

**Scheme 10:**
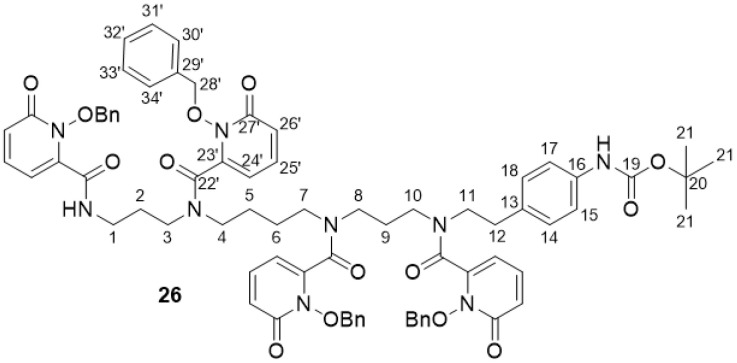
Structure of **26**.

1-(Benzyloxy)-6-oxo-1,6-dihydropyridin-2-carbonic acid **22** (0.393 g, 1.60 mmol) was dissolved in benzene (5.2 mL). Oxalyl chloride (0.25 mL, 2.90 mmol) was added dropwise under a flow of nitrogen. Every two hours, two drops of DMF were added until no more gas formation was observed (6 h). Benzene and the excess of oxalyl chloride were removed by evaporation. The obtained 1,2-HOPO acid chloride was used in the next step without further purification and was dissolved in DCM (4.7 mL). This solution was added dropwise to a mixture of **25** (0.130 g, 0.31 mmol) in DCM (3.5 mL) and a potassium carbonate solution (40%, 0.70 mL) over a period of 30 min. The mixture was stirred for 48 h at ambient temperature. In the following, the water phase was extracted with DCM (3 × 10 mL). After evaporation, the crude product was purified by semipreparative HPLC using a gradient of 10–100% MeCN + 0.1% TFA in 12 min (*R*_t_ = 6.35 min). The product was isolated as a colorless solid in a 17% yield (70.3 mg, 52.8 µmol). ^1^H-NMR (500 MHz, DMSO-*d*_6_, 25 °C) *δ* 9.37–4.76 (m, 44 H), 2.87–2.55 (m, 16H, *H*-1, *H*-3, *H*-4, *H*-7, *H*-8, *H*-10, *H*-11, *H*-12), 1.92–0.97 (m, 17H, *H*-2, *H*-5, *H*-6, *H*-9, *H*-21). ^13^C-NMR (125 MHz, DMSO-*d*_6_, 25 °C) *δ* 161.61, 161.34, 157.92, 157.65, 153.22, 143.21, 140.70, 139.38, 139.26, 138.41, 138.25, 134.28, 134.02, 130.06, 129.59, 129.49, 129.22, 128.90, 122.87, 122.41, 118.68, 104.28, 102.82, 99.13, 79.69, 79.42, 79.33, 79.16, 78.80, 46.25, 43.89, 42.28, 37.11, 36.77, 29.45, 28.58 (*C*-21), 25.46, 25.08. MALDI-MS (*m*/*z*) for [M + H]^+^ (calculated): 1330.63 (1330.58). HR-ESI-MS (*m*/*z*) for [M + H]^+^ (calculated): 1330.5817 (1330.5820), [M + Na]^+^ (calculated): 1352.5636 (1352.5639). See Scheme 10 for molecular formula.

#### 2.2.11. 3,4,3-(LI-1,2-HOPOBn)-NH_3_Cl **27**

**Scheme 11:**
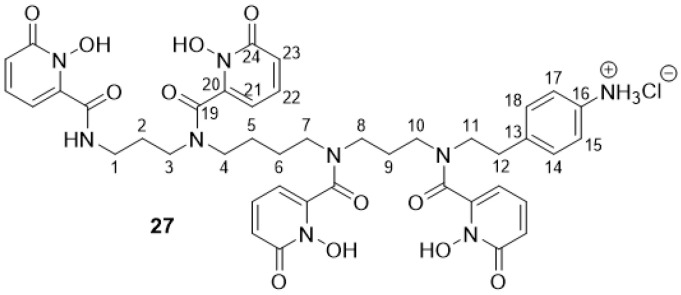
Structure of **27**.

The synthesis was established on the basis of a published procedure. 3,4,3-(LI-1,2-HOPOBn)-Ph-NH-Boc, **26** (60.0 mg, 0.045 mmol), was dissolved in dichloromethane (18.7 mL) under Ar atmosphere. A solution of BCl_3_ in *p*-xylene (1 M, 4.8 mL, 4.825 mmol) was added, and the reaction was stirred for 16 h at ambient temperature. The formed colorless precipitate was centrifuged off and washed with acetone (3 × 20 mL) and diethyl ether (3 × 20 mL). The product was obtained as a colorless solid in a 91% yield (37.2 mg, 0.041 mmol). ^1^H-NMR (500 MHz, CD_3_OD, 25 °C) *δ* 8.14–5.48 (m, 16H, *H*-14, *H*-15, *H*-17, *H*-18, *H*-21′, *H*-22′, *H*-23′), 3.80–2.78 (m, 16H, *H*-1, *H*-3, *H*-4, *H*-7, *H*-8, *H*-10, *H*-11, *H*-12), 2.13–1.29 (m, 8H, *H*-2, *H*-5, *H*-6, *H*-9). ^13^C-NMR (125 MHz, CD_3_OD, 25 °C) *δ* 163.69, 163.66, 163.61, 163.35, 162.59, 162.47, 160.41, 160.24, 142.89, 142.73, 142.68, 142.54, 142.27, 141.60, 141.10, 139.84, 139.65, 138.86, 131.87, 131.79, 131.70, 123.93, 120.51, 108.70, 108.11, 105.61, 105.50, 51.13, 49.51, 49.43, 48.35, 48.19, 47.78, 47.55, 47.30, 47.25, 46.65, 43.85, 43.73, 43.63, 38.33, 38.01, 34.69, 33.70, 33.51, 29.08, 27.86, 26.28, 25.89, 25.36. MALDI-MS (*m*/*z*) for [M + Fe − 2H]^+^ (calculated): 922.54 (923.25), [M + Fe + Na − 3H]^+^ (calculated): 944.51 (945.24). HR-ESI-MS (*m*/*z*) for [M + Fe − 2H]^+^ (calculated): 923.2522 (923.2532), [M + Fe − 4H]^−^ (calculated): 921.2387 (921.2386). See Scheme 11 for molecular formula.

#### 2.2.12. 3,4,3-(LI-1,2-HOPOBn) Tetrazine **4**

**Scheme 12:**
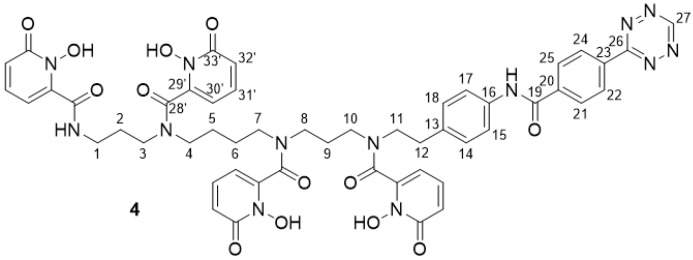
Structure of **4**.

Compound **27** (27.0 mg, 29.8 µmol) was dissolved in DMSO (0.6 mL). 4-(1,2,4,5-tetrazin-3-yl)benzoic acid (6.0 mg, 29.8 µmol) and PyBOP (31 mg, 59.6 µmol) were dissolved in DMSO (0.6 mL). The solutions were combined, and DIPEA (20.0 µL, 119.2 µmol) was added. The reaction mixture was stirred for 4 h at ambient temperature. The crude product was purified by analytical HPLC using a gradient 0–100% MeCN + 0.1% TFA for 5 min (*R*_t_ = 1.89 min). The product was obtained as a pink solid in a yield of 16% (4.9 mg, 4.6 µmol). ^1^H-NMR (700 MHz, DMSO-*d*_6_, 25 °C) *δ* 10.66 (s, 1H, *H*-27), 10.50–10.40 (m, 1H, N*H*), 8.87–8.68 (m, 1H, N*H*), 8.70–8.62 (m, 2H, *H*-22, *H*-24), 8.30–8.21 (m, 2H, *H*-21, *H*-25), 8.21–5.73 (m, 16H, *H*-14, *H*-15, *H*-17, *H*-18, *H*-30′, *H*-31′, *H*-32′), 3.32–2.65 (m, 16H, *H*-1, *H*-3, *H*-4, *H*-7, *H*-8, *H*-10, *H*-11, *H*-12), 1.97–1.17 (m, 12H, *H*-2, *H*-5, *H*-6, *H*-9). ^13^C-NMR (175 MHz, DMSO-*d*_6_, 25 °C) *δ* 166.75, 165.13, 164.63, 161.34, 161.25, 160.26, 159.61, 157.88, 157.48, 157.32, 157.30, 142.35, 142.11, 141.94, 141.90, 141.78, 141.56, 138.46, 137.86, 137.68, 137.39, 137.23, 137.19, 137.05, 137.00, 134.55, 134.52, 129.04, 128.97, 128.89, 128.73, 128.71, 127.82, 127.79, 127.33, 120.75, 120.56, 120.50, 119.38, 119.10, 119.04, 118.98, 118.87, 117.25, 108.77, 106.32, 103.69, 103.67, 103.63, 103.61, 102.18, 102.11, 101.82, 101.49, 45.88, 26.73, 25.94. MALDI-MS (*m*/*z*) for [M + H]^+^ (calculated): 1055.32 (1054.38), [M + Na]^+^ (calculated): 1076.30 (1076.36). HR-ESI-MS (*m*/*z*) for [M + Fe − 2H]^+^ (calculated): 1107.2910 (1107.2917), [M + Fe + Na − 3H]^+^ (calculated): 1129.2734 (1129.2742). See Scheme 12 for molecular formula.

### 2.3. Syntheses of Chelator Peptide Bioconjugates

#### 2.3.1. DFO-c(RGDfK) **29**

To a solution of **28** (1.00 mg, 13.2 µmol) in DMSO (50 µL) was added a solution of **1** (1.21 mg, 14.6 µmol) in DMSO (50 µL). A DPBS buffer (DPBS = Dulbecco’s Balanced Salt Solution) (10 mM, pH 7.4, 100 µL) was added, and the reaction mixture was kept at 25 °C for 30 min. The crude product was purified by semipreparative HPLC using a gradient 0–70% MeCN + 0.1% TFA in 8 min (*R*_t,pyridazine_ = 5.04 min, *R*_t,DHP_ = 5.52 min). A mixture of the oxidized (pyridazine) and non-oxidized (DHP) forms was obtained in an overall yield of 78% (1.6 mg, 1.0 µmol). DHP: MALDI-MS (*m*/*z*) for [M + H]^+^ (calculated): 1556.65 (1557.84), [M + Na]^+^ (calculated): 1579.31 (1579.82). HR-ESI-MS (*m*/*z*) for [M + H]^+^ (calculated): 1557.8403 (1557.8424), [M + 2H]^2+^ (calculated): 779.4243 (779.4249), [M − H]^−^ (calculated): 1555.8278 (1555.8283), [M − 2H]^2−^ (calculated): 777.4106 (777.4103). Pyridazine: MALDI-MS (*m*/*z*) for [M + H]^+^ (calculated): 1555.90 (1555.83), [M + Na]^+^ (calculated): 1578.02 (1577.81). HR-ESI-MS (*m*/*z*) for [M + H]^+^ (calculated): 1555.8244 (1555.8267), [M + 2H]^2+^ (calculated): 778.4161 (778.4170), [M − H]^−^ (calculated): 1553.8129 (1553.8122), [M − 2H]^2^^−^ (calculated): 776.4026 (776.4024).

#### 2.3.2. CTH36-c(RGDfK) **30**

The synthesis of **30** was carried out according to the literature [10]. DHP: MALDI-MS (*m*/*z*) for [M + H]^+^ (calculated): 1714.40 (1714.85), [M + Na]^+^ (calculated): 1736.27 (1736.84). HR-ESI-MS (*m*/*z*) [M + H]^+^ (calculated): 1714.8499 (1714.8547), [M + 2H]^2+^ (calculated): 857.9302 (857.9310), [M + Na + H]^2+^ (calculated): 868.9210 (868.9220), [M − H]^−^ (calculated): 1712.8393 (1712.8401), [M − 2H]^2−^ (calculated): 855.9165 (855.9164). Pyridazine: MALDI-MS (*m*/*z*) for [M + H]^+^ (calculated): 1712.89 (1712.84).

#### 2.3.3. DFO*-c(RGDfK) **31**

To a solution of **28** (1.00 mg, 13.2 µmol) in DMSO (50 µL) was added a solution of **3** (1.50 mg, 14.5 µmol) in DMSO (50 µL). A DPBS buffer (10mM, pH 7.4, 100 µL) was added, and the reaction mixture was kept at 25 °C for 30 min. The crude product was purified by semipreparative HPLC using a gradient 0–50% MeCN + 0.1% TFA for 8 min (*R*_t,pyridazine_ = 6.59 min, *R*_t,DHP_ = 6.80 min). A mixture of the oxidized (pyridazine) and non-oxidized (DHP) forms was obtained in an overall yield of 59% (1.37 mg, 7.79 µmol). DHP: MALDI-MS (*m*/*z*) for [M + H]^+^ (calculated): 1757.79 (1757.96), [M + Na]^+^ (calculated): 1779.28 (1779.94). HR-ESI-MS (*m*/*z*) for [M + H]^+^ (calculated): 1757.9527 (1757.9585), [M + 2H]^2+^ (calculated): 879.4819 (879.4829), [M − H]^−^ (calculated): 1755.9381 (1755.9439), [M − 2H]^2−^ (calculated): 877.4680 (877.4683). Pyridazine: MALDI-MS (*m*/*z*) for [M + H]^+^ (calculated): 1755.91 (1755.94).

#### 2.3.4. 3,4,3-(LI-1,2-HOPOBn)-c(RGDfK) **32**

To a solution of **28** (0.90 mg, 11.9 µmol) in DMSO (50 µL) was added a solution of **4** (1.38 mg, 13.1 µmol) in DMSO (50 µL). A DPBS buffer (10mM, pH 7.4, 100 µL) was added, and the reaction mixture was kept at 25 °C for 30 min. The crude product was purified by semipreparative HPLC using a gradient 0–70% MeCN + 0.1% TFA in 8 min (*R*_t,pyridazine_ = 5.44 min, *R*_t,DHP_ = 5.85 min). A mixture of the oxidized (pyridazine) and non-oxidized (DHP) forms was obtained in an overall yield of 84% (1.79 mg, 10.1 µmol). DHP: MALDI-MS (*m*/*z*) [M + H]^+^ (calculated): 1781.30 (1781.77), Pyridazine: MALDI-MS (*m*/*z*) for [M + H]^+^ (calculated): 1780.72 (1779.76), [M + Fe − 2H]^+^ (calculated): 1832.46 (1832.67). HR-ESI-MS (*m*/*z*) [M + H]^+^ (calculated): 1779.7513 (1779.7551), [M + 2H]^2+^ (calculated): 890.3807 (890.3812).

#### 2.3.5. DOTA-GA-c(RGDfK) **33**

To a solution of **28** (1.00 mg, 13.2 µmol) in DMSO (50 µL) was added a solution of **5** (0.94 mg, 14.6 µmol) in DMSO (50 µL). A DPBS buffer (10mM, pH 7.4, 100 µL) was added, and the reaction mixture was kept at 25 °C for 30 min. The crude product was purified by semipreparative HPLC using a gradient 0–70% MeCN + 0.1% TFA in 8 min (*R*_t,pyridazine_ = 4.78 min, *R*_t,DHP_ = 5.18 min). A mixture of the oxidized (pyridazine) and non-oxidized (DHP) forms was obtained in an overall yield of 70% (1.34 mg, 9.8 µmol). To obtain the pyridazine as the sole product, 1% TFA in water (100 µL) was added, and the mixture was incubated for 15 h at ambient temperature, followed by purification by semipreparative HPLC. DHP: MALDI-MS (*m*/*z*) for [M + H]^+^ (calculated): 1372.78 (1373.68). Pyridazine: MALDI-MS (*m*/*z*) for [M + H]^+^ (calculated): 1371.45 (1371.67), [M + Na]^+^ (calculated): 1393.49 (1393.65). HR-ESI-MS (*m*/*z*) [M + H]^+^ (calculated): 1371.6662 (1371.6692), [M + 2H]^2^^+^ (calculated): 686.3380 (686.3383), [M + H + Na]^2^^+^ (calculated): 697.3380 (697.3292), [M − H]^−^ (calculated): 1369.6557 (1369.6546).

### 2.4. Radiochemistry

To a solution of [^89^Zr]Zr oxalate (1 M, 40–55 MBq) in 0.1-M HCl solution (~50 µL) was added HEPES buffer (0.25 M, pH = 9, 150 µL), and the pH of the solution was adjusted to pH = 7.0–7.3 by the addition of a NaOH solution (30%, 2–4 µL). A solution of the respective chelator–c(RGDfK) conjugate (20 nmol, 20 µL) was added, and the mixture was warmed to 37 °C, and the reaction progress was monitored by radio-HPLC and, for [^89^Zr]Zr-**31** and [^89^Zr]Zr-**32**, additionally by radio-iTLC. For DFO–c(RGDfK), DFO*–c(RGDfK) and CTH36–c(RGDfK), an ^89^Zr incorporation rate of ≥96% was accomplished within 1 h. For ^89^Zr incorporation in 3,4,3-(LI-1,2-HOPO)–c(RGDfK), the mixture had to be warmed to 37 °C for 5 h to achieve 96 to 97% incorporation. The products were obtained in nonoptimized molar activities of 2–2.75 GBq/µmol.

Preparation of [^89^Zr]ZrCl_4_ (used only for labeling experiments of **5**): A Sep-Pak Accell Plus QMA Carbonate Light cartridge (Waters, 46 mg) was rinsed first with ethanol (5 mL), followed by HCl (1 M, 7.5 mL), saline solution (0.9%, 7.5 mL) and, finally, Tracepur water (7.5 mL). The [^89^Zr]Zr oxalate solution (8.9 MBq) was loaded onto the cartridge and washed with water (30 mL). [^89^Zr]ZrCl_4_ was eluted with HCl (1 M, 0.4 mL) in >95% efficiency.

### 2.5. Determination of logD_(7.4)_

To a mixture of 1-octanol (800 µL) and 0.05-M phosphate buffer (775 µL, pH 7.4), a solution of the respective ^89^Zr-labeled peptide conjugate (5µL of the before-obtained product solution, 0.8–1.2 MBq, 0.37–0.45 nmol) was added. The mixture was vigorously shaken for two minutes. Afterwards, the phases were separated by centrifugation. Two hundred microliters of each, the organic and the aqueous, phase were measured in a gamma counter. Each experiment was performed at least thrice, each in duplicate.

### 2.6. EDTA-Based ^89^Zr Complex Challenge Experiments

For each challenge experiment, three separate solutions of EDTA (14.61 mg, 50 µmol EDTA) in HEPES buffer (0.25 M, pH = 7.0, 380 µL) were prepared. The pH of the solutions was adjusted to pH 7.0 by the addition of a NaOH solution (30%, 12.5 µL), and Tracepur water (7.5 µL) was added to give a final volume of 400 µL of the EDTA solutions. To these solutions was added a solution of the respective ^89^Zr-labeled peptide conjugate (5 nmol, 9.7–13.9 MBq), and the solutions were kept at 25 °C over the course of the experiment. At predefined time points, each solution was analyzed by analytical radio-HPLC for determination of the amount of ^89^Zr transchelation. Each experiment was performed at least twice, each in triplicate.

### 2.7. DFT Calculations

The DFT calculations were all conducted as implemented in Spartan’20 (1.0.0) [21] using B3LYP [22,23,24] exchange correlation functionals, and 6-31G^*^ polarization basis sets were assigned for all elements, at which LANL2DZ [25,26] with an effective core potential was employed for the 4-d transition metal zirconium. Solvated-phase calculations (C-PCM dielectric constant = 78.30) were used as implemented using the PCMRAD keyword (PCMRAD = ZR ~2.68). The characterization of each optimized structure as the local minimum on the potential energy surface was carried out by a harmonic frequency analysis based on the second derivative.

Start geometries for the structure optimization were taken from the literature [6] and extended with respect to the new conjugation in Spartan. Only that part of the TCO reactant essential for the conjugation was added, and the rest of the biomolecule was omitted. The structure was subsequently optimized in Spartan.

## 3. Results and Discussion

### 3.1. Synthesis of the Backbone Tetrazine-Modified Chelator Analogs ***1**–**5***

**Figure 2:**
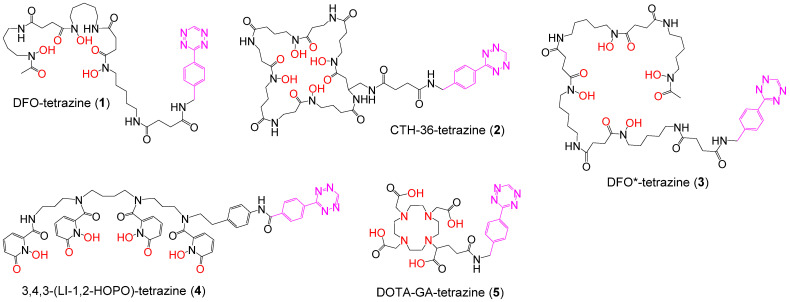
Structures of the target chelator derivatives DFO–tetrazine (**1**), CTH-36–tetrazine (**2**), DFO*–tetrazine (**3**), 3,4,3-(LI-1,2-HOPO)–tetrazine (**4**) and DOTA-GA–tetrazine (**5**). ^89^Zr-coordinating atoms are depicted in red, and the tetrazine moiety is colored in pink.

To reach the outlined aims, the syntheses of the backbone tetrazine-modified analogs of the target chelators (**1**–**5**, Figure 2) had to be established.

**Scheme 13:**
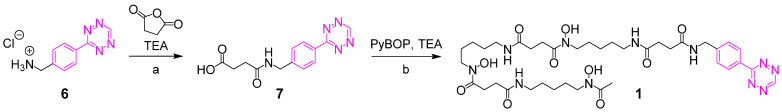
Synthesis pathway towards DFO–tetrazine **1**. Conditions: (a) 1.2 eq. succinic anhydride, 1.0 eq. TEA in DMF, 4 h, yield: 86%; (b) 1.1 eq. DFO mesylate, 2.0 eq. PyBOP, 3.0 eq. TEA in DMSO, 3 h, yield: 65%. (TEA = triethylamine).

Of these, **2 [10]** and **5 [17]** were synthesized according to literature procedures. Compound **1** was prepared following a previously described synthesis route, with some modifications in the reaction order [10] by first reacting the tetrazine amine **6** with succinic anhydride and the resulting acid **7** with DFO mesylate using PyBOP (benzotriazole-1-yl-oxy-tris-pyrrolidino-phosphonium hexafluorophosphate) as the coupling agent (Scheme 13). This route gave the product in higher yields compared to the conventional, opposite pathway reacting DFO mesylate with succinic anhydride and then conjugated the resulting acid to the tetrazine amine. The purification of **1** was initially carried out by semipreparative HPLC; however, this method resulted in a considerable loss of material and was therefore not further pursued. Instead, the product was purified by repeated precipitation from DMSO adding a 1:1 mixture of MeCN and H_2_O, giving an overall product yield of 57%.

**Scheme 14:**
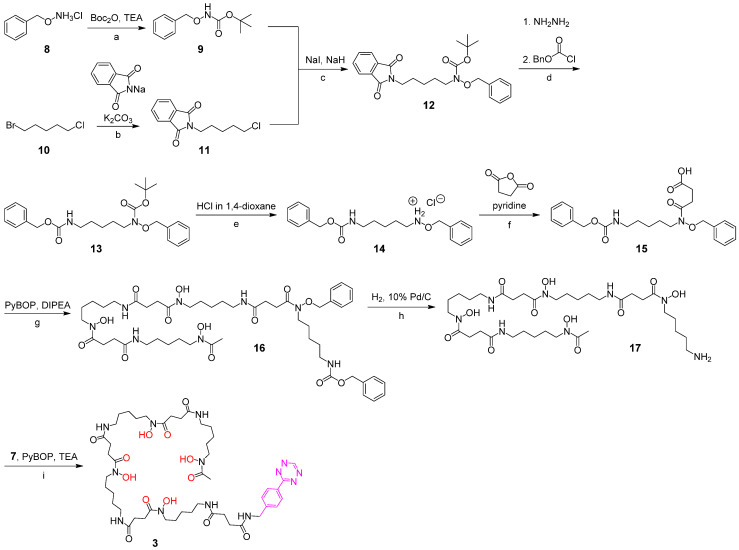
Synthesis pathway towards DFO*–tetrazine **3**. Conditions: (a) 1.0 eq. TEA, 1.0 eq. di-*tert*-butyl-dicarbonate in THF, 2.5 h, yield: 97%; (b) 1.0 eq. potassium phthalimide in DMF, 20 h, yield: 90%; (c) 0.04 eq. sodium iodide, 1.35 eq. sodium hydride in DMF, 85 °C, 19 h, yield: 95%; (d) hydrazine monohydrate in ethanol, reflux, 3 h, then 1.2 eq. Na_2_CO_3_, 1.2 eq. benzyl chloroformate in H_2_O/1,4-dioxane (2/1, *v*/*v*), 24 h, yield: 62%; (e) 4-M HCl solution in 1,4-dioxane, 4 h, yield: 79%; (f) 1.5 eq. succinic anhydride in pyridine, 12 h, yield: 79%; (g) 1.0 eq. DFO mesylate, 1.5 eq. PyBOP, 3.00 eq. DIPEA in DMF, 30 h, yield: 67%; (h) H_2_, 10% Pd/C in methanol, yield: 70%; (i) 1.0 eq. **7**, 2.0 eq. PyBOP, 3.0 eq. TEA in DMSO, 3 h, yield: 19%. (DIPEA = *N*,*N*-diisopropylethylamine).

For the synthesis of **3**, DFO* (**17**, Scheme 14) was synthesized based on a published procedure with minor changes (cf. experimental part) [7]. First, *O*-benzylhydroxylamine hydrochloride **8** was *^t^*Bu-protected to yield **9**, and 1-bromo-5-chloropentane **10** was reacted with sodium phthalimide to give **11**. The products **9** and **11** were then reacted to **12**. The exchange of the phthalimide against the Cbz-protecting group took place by deprotecting **12** first with hydrazine monohydrate, followed by the protection of the amino functionality with benzyl chloroformate, giving **13**. In the following, the *^t^*Bu-protecting group was removed using HCl in 1,4-dioxane instead of TFA (trifluoroacetic acid), yielding **14**, which was reacted with succinic anhydride to acid **15**. Compound **15** was activated using the coupling agent PyBOP instead of HATU (*N*,*N*,*N*′,*N*′-tetramethyl-*O*-(7-azabenzotriazole-1-yl)uronium hexafluorphosphate) and, in the following, reacted with DFO mesylate, giving higher conversion rates to **16** compared to HATU activation. Finally, the Cbz- and the Bn-protecting groups were removed under hydrogen atmosphere, giving DFO* **17**.

The corresponding tetrazine derivative was then obtained by reacting **17** with **7** using PyBOP as the coupling agent. The product was obtained in moderate but reproducible yields of 19% after semipreparative HPLC purification due to its low solubility in the water/acetonitrile solvent system, resulting in a considerable loss of material during purification.

Up to this point, the target chelator tetrazines **1**–**3** and **5** could be synthesized without significant difficulties, and only minor adjustments of the published reaction conditions were necessary to optimize the product yields and to obtain the backbone tetrazine-modified chelators instead of the deviant functionalized derivatives described.

**Scheme 15:**
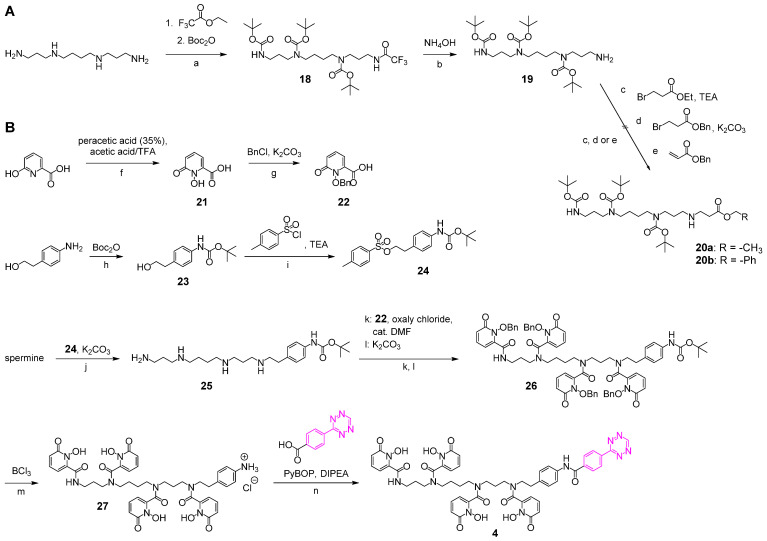
Synthesis towards 3,4,3-(LI-1,2-HOPO) tetrazine **4**. Initial, unsuccessful attempt (**A**) and optimized synthetic pathway (**B**). Conditions: (a) first, 1.0 eq. ethyl trifluoroacetate, methanol, −78 °C, 1.5 h; then, 6.0 eq. di-*t*Bu-dicarbonate, methanol, 0–23 °C, 12 h; (b) NH_4_OH, pH = 11–12, 15 h, yield over three steps: quant., contaminated with tetra-Boc-spermine; (c) R = -CH_3_: 1.1 eq. ethyl-3-bromopropanoate, 2.0 eq. TEA, acetonitrile, ambient temperature, 24 h; (d) R = -Ph: 1.1 eq. benzyl-3-bromopropanoate, 1.8 eq. K_2_CO_3_, DMF, 60 °C, 24 h; (e) R = -Ph: 1.0 eq. benzyl acrylate, methanol, ambient temperature, 2 h; (f) per-acetic acid (35%), acetic acid/TFA (1/1.5, *v*/*v*), Ar atmosphere, 23–80 °C, 17 h, yield: 69%; (g) 1.2 eq. benzyl chloride, 2.0 eq. K_2_CO_3_, methanol, reflux, 16 h, yield: 92%; (h) 2.0 eq. di-*tert*-butyldicarbonate, DCM, 16 h, yield: 96%; (i) 3.0 eq. 4-toluenesulfonyl chloride in THF, 3.3 eq. TEA, DCM, 25 h, yield: 97%; (j) 0.5 eq. 24, 2.0 eq. K_2_CO_3_, acetonitrile, 0–85 °C, reflux, 16 h, yield: 63%; (k) 5.2 eq. 22, 9.4 eq. oxalyl chloride, cat. DMF, benzene, 6 h (l) K_2_CO_3_ solution (40%), DCM, 0–23 °C, 48 h, yield over two steps: 17%; (m) BCl_3_ in *p*-xylene, DCM, 16 h, yield: 91%; (n) 1.0 eq. 4-(1,2,4,5-tetrazin-3-yl)benzoic acid, 2.0 eq. PyBOP, 3.0 eq. DIPEA, DMSO, 4 h, yield: 16%.

In contrast, the synthesis of 3,4,3-(LI-1,2-HOPO)-tetrazine, **4**, entailed considerable challenges, and several attempts were necessary to develop a successful synthesis route towards the target compound. Initially, to obtain **20a**,**b**, we followed a synthesis route towards the HOPO derivatives disclosed by Deri et al. [12] (Scheme 15A). Although the synthesis of **19** worked according to the published procedure, the purification of the product by column chromatography proved to be difficult. Besides the three-fold Boc-protected product, the four-fold protected analog was also formed, which could not be completely removed. The following reaction step applying ethyl-3-bromopropanoate, benzyl-3-bromopropanoate or benzyl acrylate to introduce the protected acid functionality into the system (**20**) also proved to be intricate, as not only the intended reaction products **20a**,**b** were formed but, also, several side products. Thus, highly complex reaction mixtures were obtained that prevented the isolation of the target substances.

In a different approach, we aimed to first introduce the tetrazine functionality into spermine by reacting spermine with *N*-(4-(1,2,4,5-tetrazin-3-yl)benzyl)-3-bromopropanamide to circumvent the problem of the following heterogeneous conversion of the amino groups, which led to inseparable product mixtures before. However, also this approach to first introduce the tetrazine into the spermine system failed due to the high basicity of the system, resulting in an instant decomposition of the tetrazine group.

Finally, another attempt was made that was based on a very recent work reported by Bhupathiraju et al., where the authors disclosed an improved synthesis route towards a 3,4,3-(LI-1,2-HOPO) isothiocyanate derivative [20]. For our purpose, the published protocol had to be adapted to obtain the target tetrazine instead of the isothiocyanate (Scheme 15B). First, 6-benzyl-6-hydroxypicolinic acid (**22**) and 4-(*t*Bu-amino)phenethyl-4-methylbenzenesulfonate (**24**) were synthesized according to the literature, giving the products in high yields between 69 and 97%. Next, spermine was reacted with **24**, resulting in a mixture of multiple products, necessitating a laborious semipreparative HPLC purification that nevertheless gave **25** in good yields of 63%. Following the published reaction pathway utilizing TEA and DMAP in DCM for the next reaction step of **25** with **22** to yield **26**, no product formation could be detected. Instead, **26** could be obtained using a small amount of aqueous K_2_CO_3_ solution in DCM; however, this also necessitated a purification by semipreparative HPLC due to the large amount of side products formed (mainly three-fold-reacted intermediates instead of four-fold-reacted products and others), limiting the product yield to only 17%. The benzyl- and *^t^*Bu-protecting groups were subsequently removed by treatment with BCl_3_ in *p*-xylene, and the obtained intermediate **27** was reacted directly without further purification with 4-(1,2,4,5-tetrazin-3-yl)benzoic acid, having been activated using PyBOP to give the target tetrazine-modified chelator **4** in moderate yields of 16%.

### 3.2. Investigation of the Properties of the Different ^89^Zr Complexes by DFT Calculations

A very recent study reported on the thermodynamic stability of several zirconium complexes by DFT calculations, giving very encouraging results for the complex formation of CTH36 and DFO* with Zr^4+^ [6]. As these calculations were conducted omitting any backbone functionalization of the chelating agents and the complex geometry was not discussed, we performed DFT calculations for the Zr complexes of the chelating agents investigated in this work and used the aforementioned data as the starting geometries. For these calculations, the chelator tetrazines **1**–**5** were conjugated to a simple model TCO to mimic the molecular situation in their respective ^89^Zr-labeled biomolecules. In Table 1, an enlargement of the optimized geometry of the complexes and the relevant bond lengths can be found. Full structures and atom coordinates can be found in the Supplementary Materials.

Overall, the calculated values of the optimized structures are in very good agreement with the literature, and in almost all cases, the backbone modification of the chelators did not show a detectable influence on the complex geometry. The only exception was Zr–DOTA–GA, which showed elongated Zr–N bonds compared to the Zr–DOTA complex. The effect of the zirconium not being in the center of the complex cavity but considerably closer to the plane of the oxygen atoms than to that of the nitrogen atoms might be an indicator of hindered Zr–DOTA–GA complex formation and a less inert complex.

Overall, there was no evidence that the kinetic inertness of one of the studied Zr complexes might be significantly compromised by the introduction of the conducted backbone functionalization and further TCO modification.

### 3.3. Syntheses of Chelator Bioconjugates ***29**–**33***

As the aim of the present study was to determine the relative kinetic inertness of the ^89^Zr complexes of the studied chelating agents, the chelator tetrazines **1**–**5** had to be introduced into a model biomolecule. The reason for this is that the kinetic inertness of the conjugated ^89^Zr complexes is much more relevant than that of the tetrazines, as the same molecular situation of conjugated complexes is present in in vivo imaging applications. In addition, the bioconjugation ability of the developed chelator tetrazines had to be demonstrated as well.

The peptide c(RGDfK), which binds to integrin α_v_β_3_ with high affinity [27] and is consequently a valuable bioactive agent accumulating in many human tumors, was chosen as clinically relevant model biomolecule for chelator introduction and ^89^Zr-labeling of the resulting conjugates. c(RGDfK) possesses—especially compared to antibody molecules—the important advantage that it exhibits a limited size and structural complexity, making the bioconjugation and the following ^89^Zr-radiolabeling reaction of the conjugates, as well as the determination of the relative kinetic inertness of the formed ^89^Zr complexes, easy to analyze and follow.

**Scheme 16:**
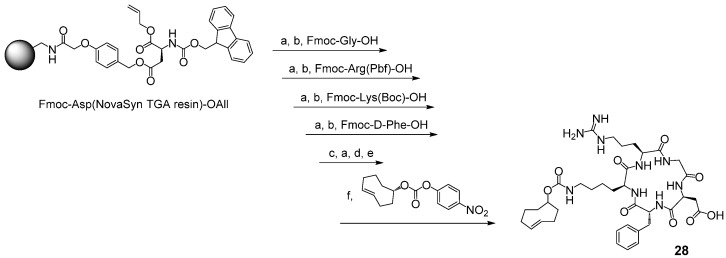
Schematic depiction of the synthesis pathway towards c(RGDfK)-TCO (**28**). Conditions: (a) Fmoc deprotection: piperidine: DMF (1:1 *v*/*v*), 2 + 5 min; (b) amino acid activation: 3.9 eq. HBTU, 4.0 eq. DIPEA, DMF, 2 min; coupling: 60 min; (c) Allyl deprotection: 0.25 eq. Pd(PPh_3_)_4_, 24 eq. phenyl silane, DCM, 3 × 30 min; (d) cyclization: 1.0 eq. HBTU, 1.0 eq. DIPEA, DMF, 16 h; (e) cleavage from resin: TFA:TIS:H_2_O (95:2.5:2.5, *v*/*v*/*v*); yield over 5 steps: 59%; (f) TCO modification: 1.0 eq. TCO active ester, 2.0 eq. DIPEA, DMF, 6 h, yield: 50%.

Thus, a complementarily functionalized c(RGDfK) peptide **28** (Scheme 16) was synthesized, carrying a *trans*-cyclooctene (TCO) unit for efficient click reaction with chelator tetrazines **1**–**5**. The cyclic peptide c(RGDfK) was built on solid support by standard Fmoc-based solid-phase peptide synthesis [28,29] and modified with TCO in solution after cleavage from the resin by reaction with the corresponding *p*-nitrophenyl active ester, yielding c(RGDfK)-TCO, **28** [10].

**Scheme 17:**
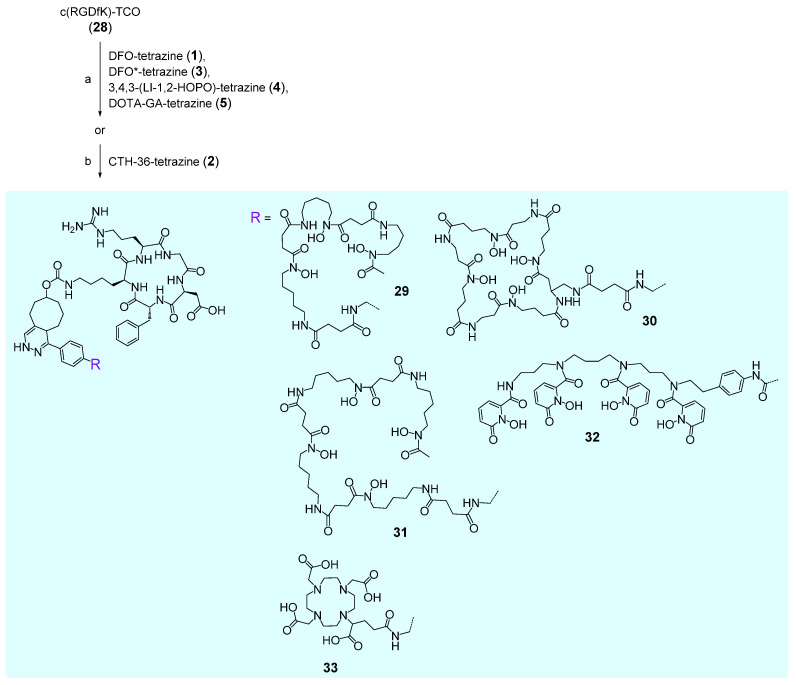
Schematic depiction of the synthesis pathway towards the chelator–peptide conjugates **29**–**33**. Conditions: (a) 1.1 eq. chelator tetrazine (**1**, **3**–**5**), DMSO/H_2_O (1/1, *v*/*v*), yields: 78% (**29**), 59% (**31**), 84% (**32**) and 70% (**33**); (b) 1.4 eq. **2**, DMSO, 65% (**30**).

Compound **28** was reacted using iEDDA click chemistry with the chelator tetrazines **1**–**5** to obtain the respective bioconjugates **29**–**33** (Scheme 17). Due to the limited solubility of **1**–**5**, the bioconjugation reactions were carried out in aqueous DMSO (**1**, **3**–**5**) or DMSO alone (**2**). All reactions were complete within minutes (obvious from disappearance of the pink tetrazine-associated color and nitrogen gas development) and gave the products in good yields of 59–84% after purification.

**Scheme 18:**
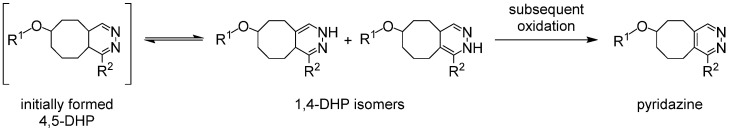
Schematic depiction of the subsequent oxidation of iEDDA-formed 4,5-dihydropyridazines to aromatic pyridazines.

It was observed during all iEDDA-based bioconjugation reactions that a side product whose amount varied among the different reactions was formed in addition to the 4,5-dihydropyridazines (DHP). When possible, the respective byproducts were isolated and analyzed by mass spectrometry, indicating the oxidization of the DHPs to their respective aromatic pyridazine systems (Scheme 18). This is in accordance with literature reports where the spontaneous oxidation of iEDDA-formed DHPs to aromatic pyridazines has been described [30,31,32].

Furthermore, we calculated the bond lengths of the formed heteroatom-containing rings by the previously mentioned DFT calculations, finding bond lengths corresponding to aromatic pyridazine (Table 2), supporting the theory of spontaneous oxidation of the formed DHPs to the respective pyridazines.

While relatively low amounts of pyridazine were formed in the reactions forming **29** and **30** (<7%), higher rates were found in the reactions towards **31**–**33** (>35%). Attempts to force the reactions into the formation of the pyridazines by acidification of the product solutions [30,34] were only successful for **33**, whereas, in all the other cases (**29**–**32**), the pH-driven formation of the pyridazine was slow compared to the formation of decomposition products. Of the latter, only **32** could be isolated as pure pyridazine by HPLC, whereas, in the other cases (**29**–**31**), the DHP and the pyridazine forms were completely inseparable. For this reason, these bioconjugates were used as mixtures for the following ^89^Zr-radiolabeling reactions, containing 4.5% (**29**), 6.2% (**30**) and 38.3% (**31**) pyridazine, respectively.

### 3.4. ^89^Zr-Radiolabeling of the Bioconjugates ***29**–**33*** and Determination of the logD_(7.4)_s of [^89^Zr]Zr-***29***–[^89^Zr]Zr-***32***

The peptide–chelator conjugates **29**–**33** were in the following radiolabeled with ^89^Zr. For **29**–**32**, the radiolabeling reactions were performed at 37 °C, whereas the ^89^Zr-labeling of DOTA was described to take 45 min at 90 °C to completion [13].

First, attempts were made to radiolabel the bioconjugates **29**–**33** using these standard reaction conditions. For this purpose, an amount of 20 nmol of the respective radiolabeling precursor **29**–**32** was incubated with 40–55 MBq of [^89^Zr]Zr oxalate solution at pH 7.0 in buffered solution at 37 °C, and the complexation progress was monitored by analytical radio-HPLC.

**Figure 3:**
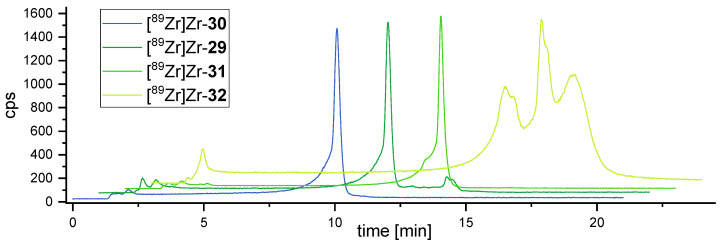
Analytical radio-HPLC chromatograms of the reaction mixtures of [^89^Zr]Zr-**29**–[^89^Zr]Zr-**32**.

After one hour, an ^89^Zr incorporation rate of ≥96% was observed for **29**–**31**, demonstrating the fast ^89^Zr complex formation of **29**–**31**. Under the used radiolabeling conditions, the mixtures of DHPs and pyridazines, which were used in the case of **29**–**31** (vide supra), homogenized, giving the oxidized pyridazines. For **31**, this homogenization process was complete within the first hour of radiolabeling, whereas it took an additional one or three hours for **30** and **29**, respectively, to form the uniform pyridazine products. The products [^89^Zr]Zr-**29**–[^89^Zr]Zr-**31** were obtained in a nonoptimized molar activity of 2–2.75 GBq/µmol in the form of a single product peak during radio-HPLC (Figure 3).

In contrast, the ^89^Zr incorporation into **32** was considerably less effective, requiring prolonged reaction times of 5 h for sufficient ^89^Zr complexation of ≥96% under the same conditions. Furthermore, [^89^Zr]Zr-**32** could not be obtained in form of a single product, although the pure pyridazine was applied as the precursor (Figure 3). This effect, that more than one product peak is formed during ^89^Zr-radiolabeling of 3,4,3-(LI-1,2-HOPO), has been described before, and it was assumed that this could be a result of an initial formation of a kinetically favored product that is, over time, converted into a thermodynamically favored one [11]. This reasonable assumption is, however, not able to explain the observed formation of three separate peaks observed here. A possible explanation could be that different structure conformers of the same complex are formed, an effect being further enhanced by the backbone functionalization of the chelator. This effect would not be observable during antibody labeling. In a highly complex system like an antibody, a small molecular change has no influence on the retention time on conventional or size exclusion HPLC. To evaluate even small structural differences, it is an advantage to investigate a peptide of lower molecular weight, where even small changes in the structure result in different HPLC retention times. The formation of different ^89^Zr complexation products is, of course, of particular interest in terms of the kinetic inertness, since different conformers might exhibit different stabilities. If this were the case, it could limit the applicability of 3,4,3-(LI-1,2-HOPO) for stable 89Zr introduction.

The ^89^Zr-radiolabeling of **33** was initially also tested using the mild reaction conditions mentioned above, but no ^89^Zr incorporation could be observed at 37 °C, so the temperature was increased to 99 °C for several hours. However, even under these harsh conditions, no incorporation of the radiometal could be detected, and all ^89^Zr^4+^ was present in the free form. Thus, different pH values (pH 4, 5 and 9) of the reaction solutions were tested, but this did not result in any radiometal incorporation into the chelator either. As Pandya et al. reported that the use of [^89^Zr]ZrCl_4_ instead of [^89^Zr]Zr oxalate solution resulted in considerably better ^89^Zr uptake into DOTA [13], we also used [^89^Zr]ZrCl_4_ for our labeling attempts on **33**. However, this change did not result in any ^89^Zr incorporation. In principle, this effect could be a result of the hydrolysis of ^89^Zr^4+^ to [Zr_4_(OH)_8_(OH_2_)_16_]^8+^, which is much more likely to occur after exchange of the stabilizing oxalate by chloride ions and is accelerated by low chloride concentrations and long standing times at high pH at elevated temperatures, limiting the apparent molar activities during ^89^Zr–DOTA labeling [35]. As we, however, used relatively high chloride concentrations of 1 mol/L and directly used ^89^Zr^4+^ after the counter ion exchange at different pH values (among these acidic conditions), it seems to be unlikely that this is the reason for the observed missing ^89^Zr incorporation, especially as we did not see any activity uptake by the chelator at all and not only to a limited extent.

An alternative to the use of DOTA–GA for biomolecule modification and ^89^Zr labeling would, of course, be the use of the corresponding DOTA tetrazine without an additional backbone functionalization and carboxylic group, but its use would raise the question of the inertness of the complex formed, as one of the carboxylates actually responsible for ^89^Zr coordination was, in this case, used for conjugation, forming an acid amide. This acid amide, however, would most probably be less suitable for radiometal coordination compared to the hard oxygen atoms of the carboxylic group, resulting in a considerably reduced inertness of the resulting complex, as has already been shown in the example of Cu^2+^ and its corresponding chelators [36,37].

In the following, the log*D*_(7.4)_s of the labeled agents [^89^Zr]Zr-**29**–[^89^Zr]Zr-**32** were determined in order to assess the influence of the respective chelator on the overall compound lipophilicity. A high lipophilicity of the chelating agent could, e.g., result in a considerable plasma protein binding and, thus, in an unspecific background, as well as liver uptake of a correspondingly modified biomolecule, affecting the target visualization with PET [38,39,40,41].

The log*D*_(7.4)_ values of the ^89^Zr-labeled peptide–chelator conjugates were determined by their distribution coefficient between n-octanol and phosphate buffer at pH 7.4. The lipophilicity was determined to decrease in the order from [^89^Zr]Zr-**32** (log*D*_(7.4)_: −1.76 ± 0.08) over [^89^Zr]Zr-**29** (log*D*_(7.4)_: −2.32 ± 0.21) and [^89^Zr]Zr-**31** (log*D*_(7.4)_: −2.49 ± 0.03) to [^89^Zr]Zr-**30** (log*D*_(7.4)_: −2.77 ± 0.18). Compared to the three nonaromatic chelating agents exhibiting a high hydrophilicity, the poly-aromatic 3,4,3-(LI-1,2-HOPO) system shows a relatively high lipophilicity. Although this should be considerably less relevant for the modification and labeling of antibodies compared to peptides, the use of 3,4,3-(LI-1,2-HOPO) could nevertheless result in a somewhat higher background accumulation compared to the other chelating agents DFO, DFO* and CHT-36, which could then be explained, at least in part, by the higher lipophilicity of the system [41].

### 3.5. Comparative Assessment of the Relative Kinetic Inertness of the Complexes [^89^Zr]Zr-***29***–[^89^Zr]Zr-***32*** by Challenge Experiments

In order to directly compare and assess the kinetic inertness of the formed ^89^Zr complexes of [^89^Zr]Zr-**29**–[^89^Zr]Zr-**32**, we performed complex challenge experiments using EDTA as the challenging agent of [^89^Zr]Zr-**30**–[^89^Zr]Zr-**32** in comparison to [^89^Zr]Zr-**29** serving as the gold standard. Although a complex challenge experiment cannot provide information about the absolute inertness of a complex under in vivo conditions, it does allow the determination of the relative inertness of different complexes and therefore represents the standard method for the in vitro investigation of complex stability, mimicking the challenge of a radiometal complex by different endogenous substances being present in extremely high excess under in vivo imaging conditions.

**Figure 4:**
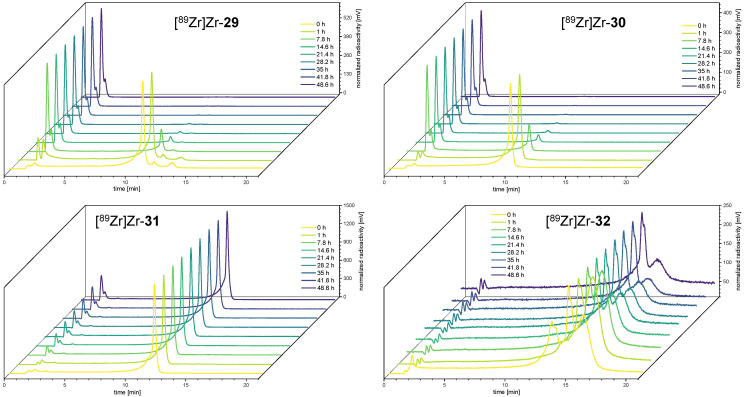
Exemplary analytical radio-HPLC chromatograms over the course of the complex challenge experiments for the different ^89^Zr complexes investigated (5 nmol of each ^89^Zr complex ([^89^Zr]Zr-**29**–[^89^Zr]Zr-**32**) were incubated with 50 µmol of EDTA, and the transfer of ^89^Zr to EDTA was monitored over 48 h).

**Figure 5:**
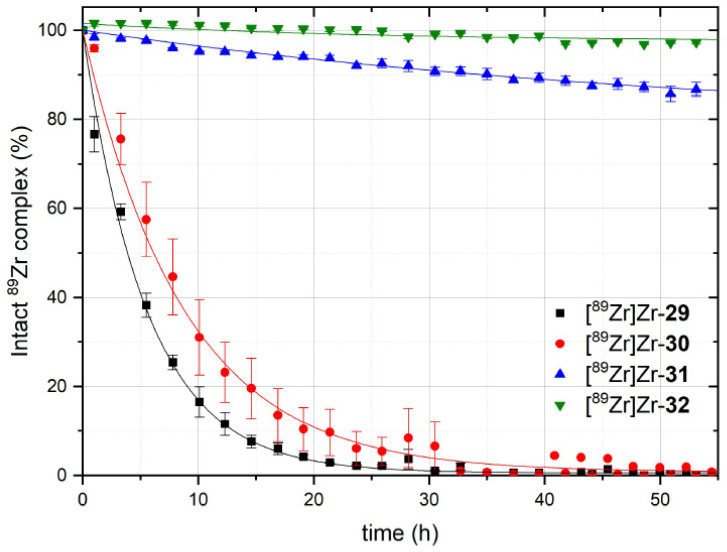
Summary of the results of the complex challenge experiments of [^89^Zr]Zr-**29**–[^89^Zr]Zr-**32** using a 10,000-fold excess of EDTA as the challenging agent. The radiochemical purity of the starting materials was normalized to 100% at the start of the EDTA challenge.

Since it was expected that all complexes to be studied (except [^89^Zr]Zr-DFO) would exhibit a high inertness against the challenge, the experiments were performed using a very high excess of 10,000 eq. of EDTA as the challenging agent. The transchelation was monitored for up to 54 h by analytical radio-HPLC and showed significant differences between the different complexes (Figure 4 and Figure 5).

In this context, the ^89^Zr–DFO complex of [^89^Zr]Zr-**29** showed the expected limited inertness and rapid transfer of the radiometal to EDTA.

Surprisingly, the ^89^Zr–CTH-36 complex of [^89^Zr]Zr-**30** showed a slightly higher but still relatively low inertness and an associated rapid transfer of the ^89^Zr, while the ^89^Zr–DFO* complex of [^89^Zr]Zr-**31** and the ^89^Zr-3,4,3-(LI-1,2-HOPO) complex of [^89^Zr]Zr-**32** demonstrated a high resistance to the challenge. The poor performance of CTH-36 was very astonishing, since it should actually form stable complexes with ^89^Zr^4+^ on the basis of preliminary studies [10] and, also, theoretical considerations, which were also reflected in the very good results of recent DFT calculations on the thermodynamic stability of its ^89^Zr complex [6].

However, in comparison, DFO* and 3,4,3-(LI-1,2-HOPO) exhibited a significantly better—and comparably high—kinetic stability of their respective ^89^Zr complexes. This clearly demonstrates that the determination of thermodynamic stability allows, as expected, only very limited conclusions to be drawn about the actual suitability of a chelating agent for the formation of kinetically inert complexes.

Due to the results found here, the latter two compounds would be ideal candidates to study in terms of the inertness of their ^89^Zr complexes in vivo, whereas CTH-36 and DOTA-GA seem to be unsuitable for ^89^Zr introduction.

We are planning the in vivo evaluation of **3** and **4** using suitably modified antibodies. For this, the determination of the immunoreactivity of the obtained conjugates, ^89^Zr radiolabeling and then investigating the in vivo pharmacokinetics of the radioligands in direct comparison over a timespan of several days will follow shortly. Of particular interest would be the extent to which the significantly higher lipophilicity of **4** affects the biodistribution of the respectively modified antibody and, also, whether the different complex species formed during radiolabeling using 3,4,3-(LI-1,2-HOPO) all exhibit a high kinetic inertness or if more advantageous in vivo pharmacokinetics will be found for the [^89^Zr]Zr–DFO*-modified antibody.

## 4. Conclusions

The results shown indicate that the chelator tetrazines **1**–**5** developed here are very well-suited to efficiently functionalize biomolecules and that **1**–**4** are applicable for the radiolabeling of biologically relevant agents with ^89^Zr.

By means of DFT calculations, it could be demonstrated that the backbone modifications of the developed chelates do not negatively affect the complex geometry and, thus, the radiolabeling of the chelator cores (at least apart from the DOTA–GA chelate).

The radiolabeling of the chelator–peptide bioconjugates with ^89^Zr revealed some significant differences between the chelating agents: While the DFO, CTH-36 and DFO* bioconjugates exhibited very favorable ^89^Zr-radiolabeling properties and advantageously high hydrophilicities of the labeled biomolecules, the 3,4,3-(LI-1,2-HOPO) peptide showed a considerably lower ^89^Zr-radiolabeling efficiency, and the formation of an inhomogeneous labeling product of considerably higher lipophilicity could be validated.

The determination of the kinetic inertness of the formed ^89^Zr complexes revealed a low stability of the [^89^Zr]Zr–DFO complex but, surprisingly, also an unexpected considerable lability of the [^89^Zr]Zr–CTH-36 complex. Only [^89^Zr]Zr–DFO* and [^89^Zr]Zr–3,4,3-(LI-1,2-HOPO) proved a high inertness against the competition challenge experiments, illustrating that a high thermodynamic stability of a complex is—as expected, but sometimes implied otherwise—not a good predictor of the inertness of a radiometal complex.

In subsequent studies, **3** and **4** will be investigated in direct comparison under in vivo conditions (including introduction into an IgG antibody, radiolabeling with ^89^Zr and determination of the immunoreactivity of the conjugates and application in an appropriate disease model, monitoring the in vivo pharmacokinetics over several days) to finally identify the most suitable chelating agent for future clinical applications.

## Figures and Tables

**Table 1:** Enlargement of the DFT-optimized geometry of the Zr complexes of **1**–**5**; relevant bond lengths of Zr-**1**–Zr-**5** and literature data for Zr–DFO(H_2_O)_2_, Zr–CTH-36, Zr–DFO*, Zr–3,4,3-(LI-1,2-HOPO) and Zr–DOTA.

	Optimized Structure	Calculated Bond Lengths [Å]	Literature Values for Bond Lengths [Å]	
product of Zr-**1** and TCO-butyl carbamate	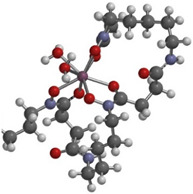		Zr-DFO(H_2_O)_2_ [6]	
		2.2260 (Zr-O (C=O))	2.2317	
		2.1563 (Zr-O (N-O))	2.1736	
		2.2232 (Zr-O (C=O))	2.2305	
		2.1321 (Zr-O (N-O))	2.1447	
		2.1899 (Zr-O (C=O))	2.1993	
		2.1829 (Zr-O (N-O))	2.2023	
		2.4080 (Zr-O (H_2_O))	2.4335	
		2.3523 (Zr-O (H_2_O))	2.3656	
product of Zr-**2** and TCO-butyl carbamate	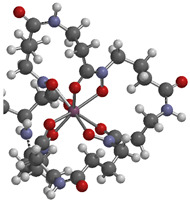		Zr-CTH-36 [10]	Zr-CTH-36 [6]
		2.2749 (Zr-O (C=O))	2.3250	2.2862
		2.1746 (Zr-O (N-O))	2.2411	2.1979
		2.2787 (Zr-O (C=O))	2.3250	2.2862
		2.1780 (Zr-O (N-O))	2.2411	2.1978
		2.2778 (Zr-O (C=O))	2.3250	2.2862
		2.1816 (Zr-O (N-O))	2.2411	2.1979
		2.2871 (Zr-O (C=O))	2.3250	2.2865
		2.1714 (Zr-O (N-O))	2.2411	2.1979
product of Zr-**3** and TCO-methyl carbamate	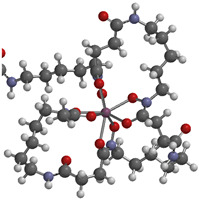		Zr-DFO* [6]	
		2.2454 (Zr-O (C=O))	2.2549	
		2.2079 (Zr-O (N-O))	2.2241	
		2.3099 (Zr-O (C=O))	2.3216	
		2.1475 (Zr-O (N-O))	2.1651	
		2.2533 (Zr-O (C=O))	2.2945	
		2.2345 (Zr-O (N-O))	2.2289	
		2.2853 (Zr-O (C=O))	2.3249	
		2.1845 (Zr-O (N-O))	2.1954	
product of Zr-**4** and TCO-butyl carbamate	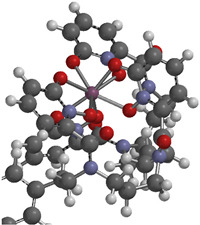		Zr-3,4,3-(LI-1,2-HOPO) [11]	Zr-3,4,3-(LI-1,2-HOPO) [6]
		2.2446 (Zr-O (C=O))	2.2394	2.2316
		2.2043 (Zr-O (N-O))	2.2876	2.2574
		2.2099 (Zr-O (C=O))	2.2714	2.2410
		2.2094 (Zr-O (N-O))	2.2324	2.2139
		2.2528 (Zr-O (C=O))	2.2820	2.2765
		2.2173 (Zr-O (N-O))	2.2258	2.2079
		2.1734 (Zr-O (C=O))	2.1979	2.1871
		2.3182 (Zr-O (N-O))	2.3215	2.3012
product of Zr-**5** and TCO-butyl carbamate	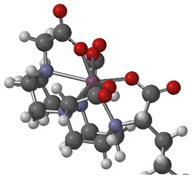		Zr-DOTA [13]	Zr-DOTA [6]
		2.5652 (Zr-N)	2.415	2.5470
		2.1107 (Zr-O)	2.133	2.1570
		2.4766 (Zr-N)	2.415	2.4952
		2.1655 (Zr-O)	2.133	2.1690
		2.5244 (Zr-N)	2.415	2.5471
		2.1207 (Zr-O)	2.133	2.1569
		2.4762 (Zr-N)	2.415	2.4952
		2.1595 (Zr-O)	2.133	2.1690

**Table 2:** Bond lengths in the formed heterocycles obtained by DFT calculations, as well as the literature data for free pyridazine [33] given for comparison. All values are given in Å.

	N(1)-N(2)	N(1)-C(1)	C(1)-C(2)	C(2)-C(3)	C(3)-C(4)	C(4)-N(2)
product of Zr-**1** and TCO-butyl carbamate	1.334	1.346	1.420	1.400	1.402	1.332
product of Zr-**2** and TCO-butyl carbamate	1.337	1.330	1.403	1.398	1.420	1.343
product of Zr-**3** and TCO-methyl carbamate	1.335	1.331	1.403	1.400	1.421	1.345
product of Zr-**4** and TCO-butyl carbamate	1.333	1.345	1.418	1.400	1.402	1.332
product of Zr-**5** and TCO-butyl carbamate	1.334	1.345	1.422	1.400	1.403	1.331
pyridazine	1.337	1.338	1.400	1.385	1.400	1.338

## Data Availability

The data is contained within the article and the Supplementary Materials.

## Supplementary Materials

The following are available online at https://www.mdpi.com/article/10.3390/cancers13246349/s1, NMR spectra of **1**, **3**, **4**, **26** and **27**. DFT-optimized complex geometries of Zr-**1**–Zr-**5**, reacted with model TCO and the corresponding cartesian xyz coordinates, and the results of radio-iTLC analyses of [^89^Zr]Zr-**31** and [^89^Zr]Zr-**32**.
