# Supplementary material for: Side-by-Side Comparison of Five Chelators for 89Zr-Labeling of Biomolecules: Investigation of Chemical/Radiochemical Properties and Complex Stability
Source: Cancers (Basel). 2021 Dec 17;13(24):6349. doi: 10.3390/cancers13246349 (PMC8699488; doi:10.3390/cancers13246349)
Supplement: Supplementary file 1 [file cancers-13-06349-s001.zip › cancers-1473613-supplementary.pdf]

cancers

MDPI

Supplementary Materials

Side-By-Side Comparison of Five Chelators for ^{89}Zr-Labeling of Biomolecules: Investigation of Chemical/Radiochemical Properties and Complex Stabi

Helen Damerow, Ralph Hübner, Benedikt Judmann, Ralf Schirrmacher, Björn Wängler, Gert Fricker and Carmen Wängler

File S1. NMR Spectra, results of DFT calculations and radio-iTLC analyses

Content

NMR spectra of **1**, **3**, **4**, **26** and **27**	pages 2–6
DFT-optimized complex geometries of Zr-**1** – Zr-**5**, reacted with model TCO and corresponding cartesian xyz-coordinates	pages 7–20
Results of radio-iTLC analyses of [^{89}Zr]Zr-**31** and [^{89}Zr]Zr-**32**	page 21

NMR spectra

DFO tetrazine ***1***

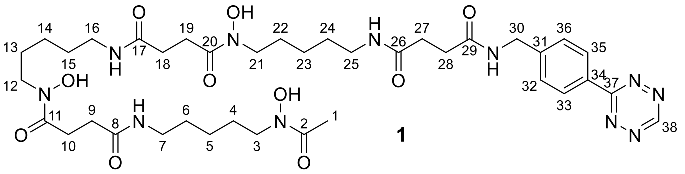

1

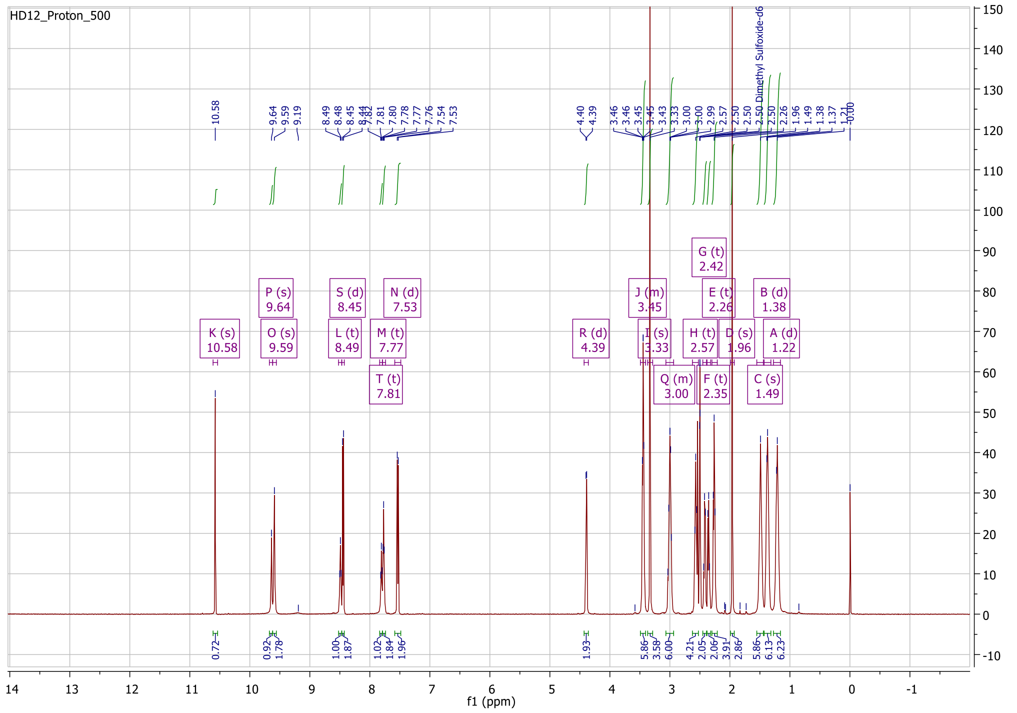

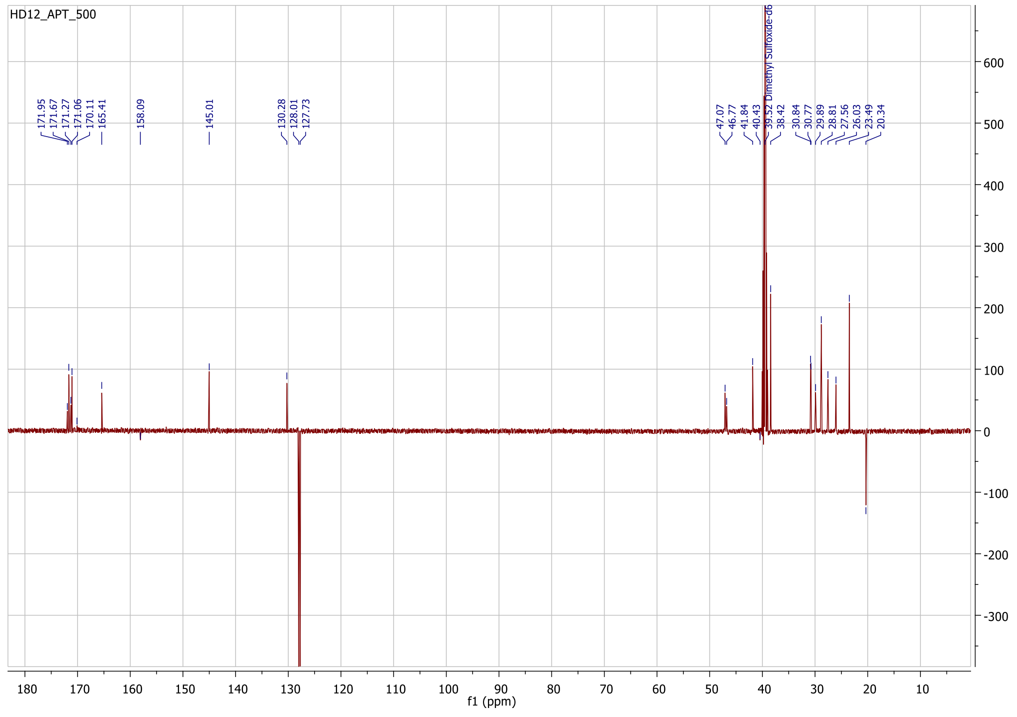

DFO tetrazine* **3**

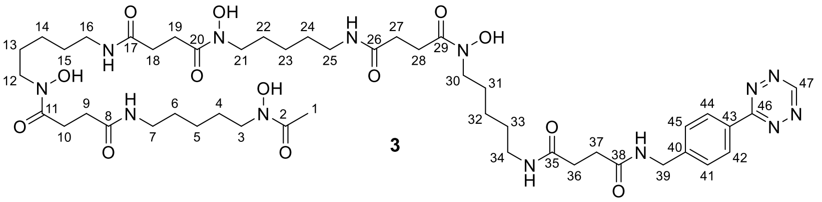

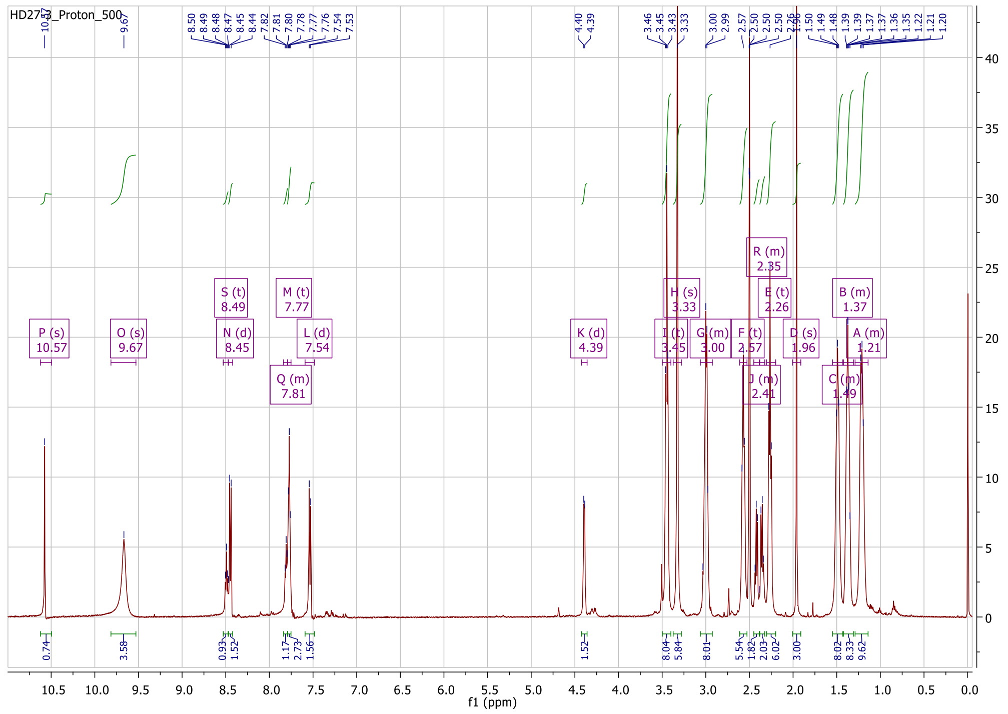

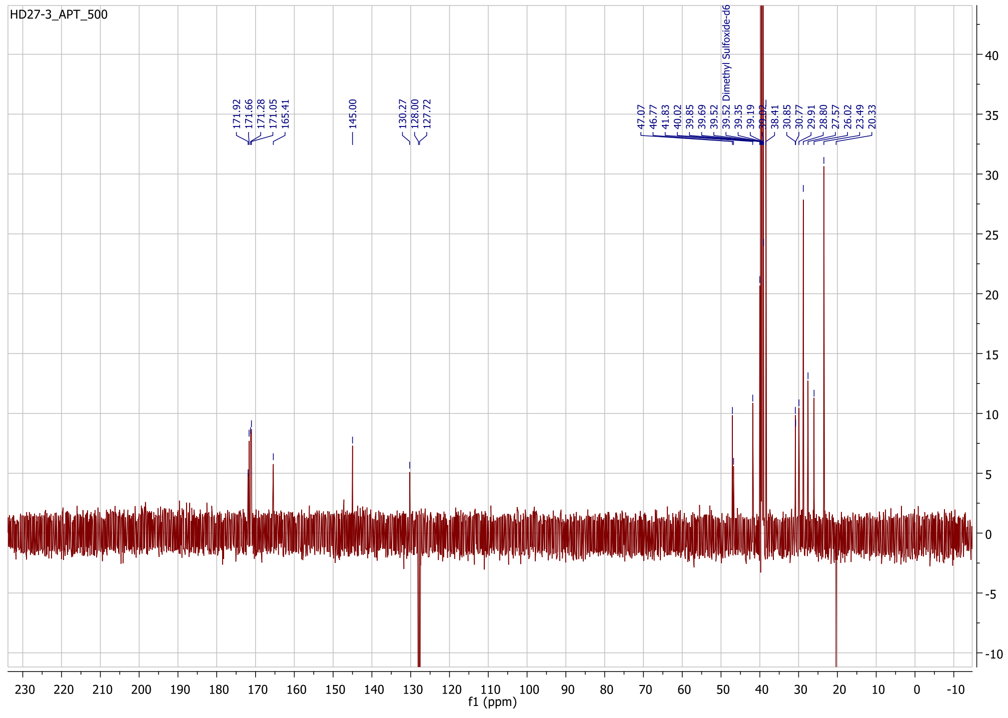

3,4,3-(LI-1,2-HOPOBn)-Ph-NH-Boc **26**

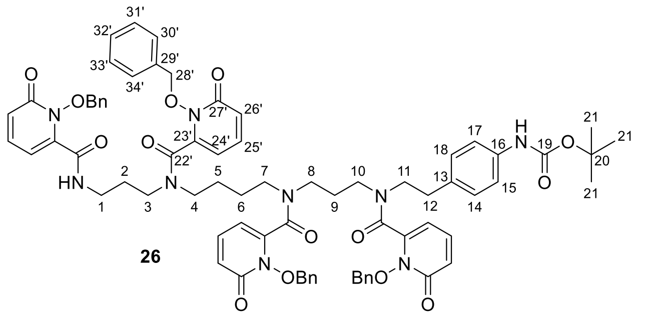

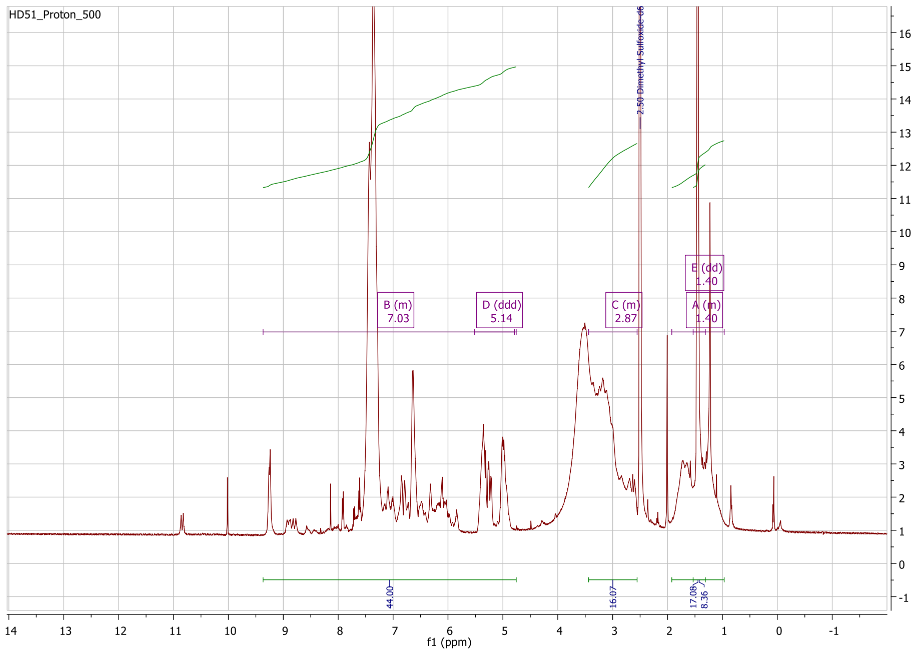

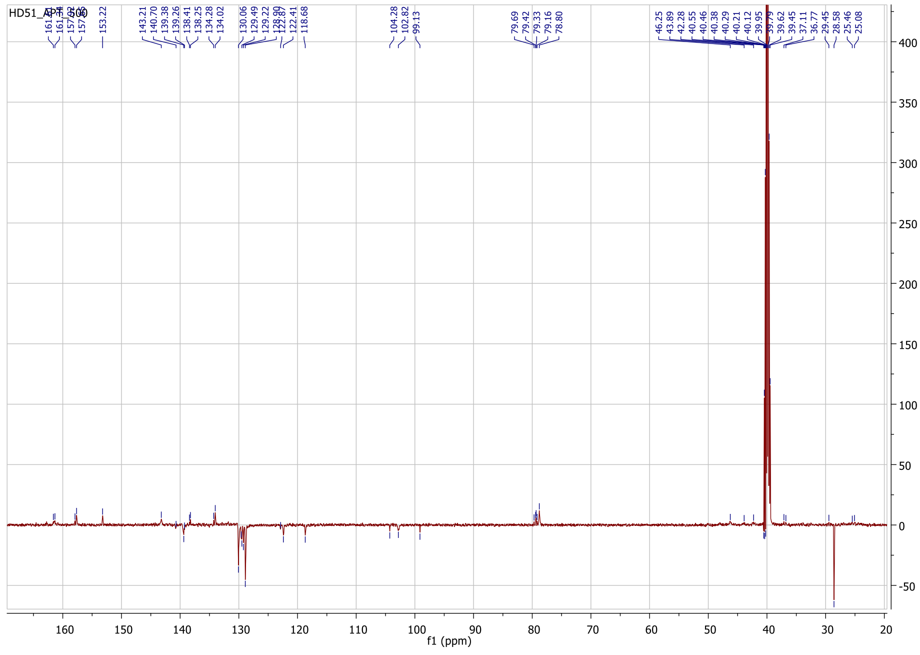

3,4,3-(LI-1,2-HOPOBn)-Ph-NH$_3$Cl **27**

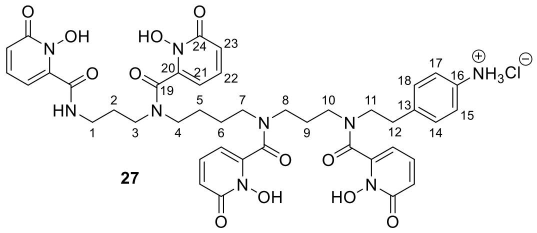

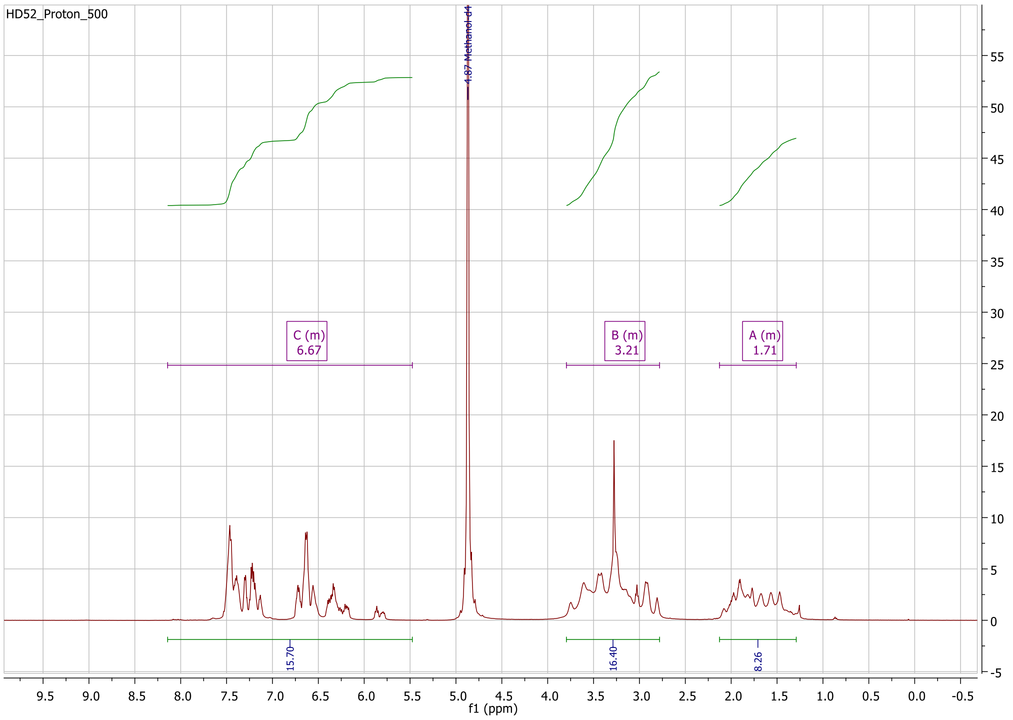

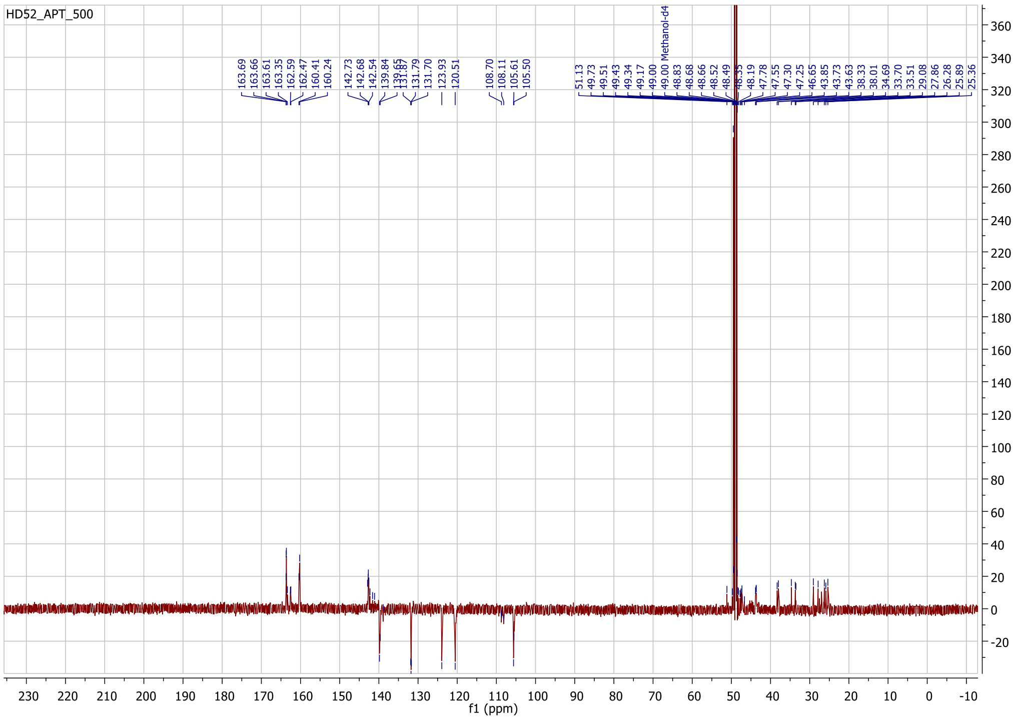

3,4,3-(LI-1,2-HOPOBn)-Ph tetrazine **4**

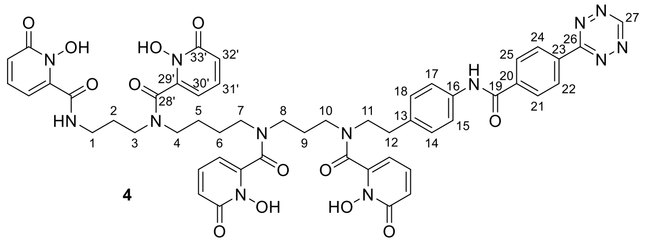

4

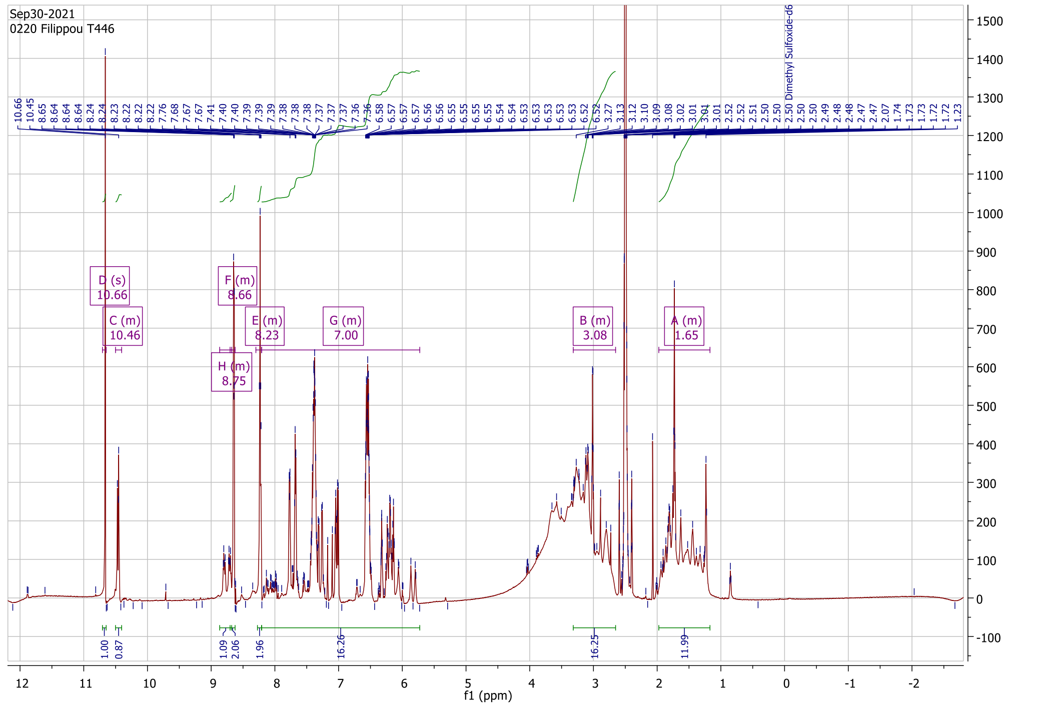

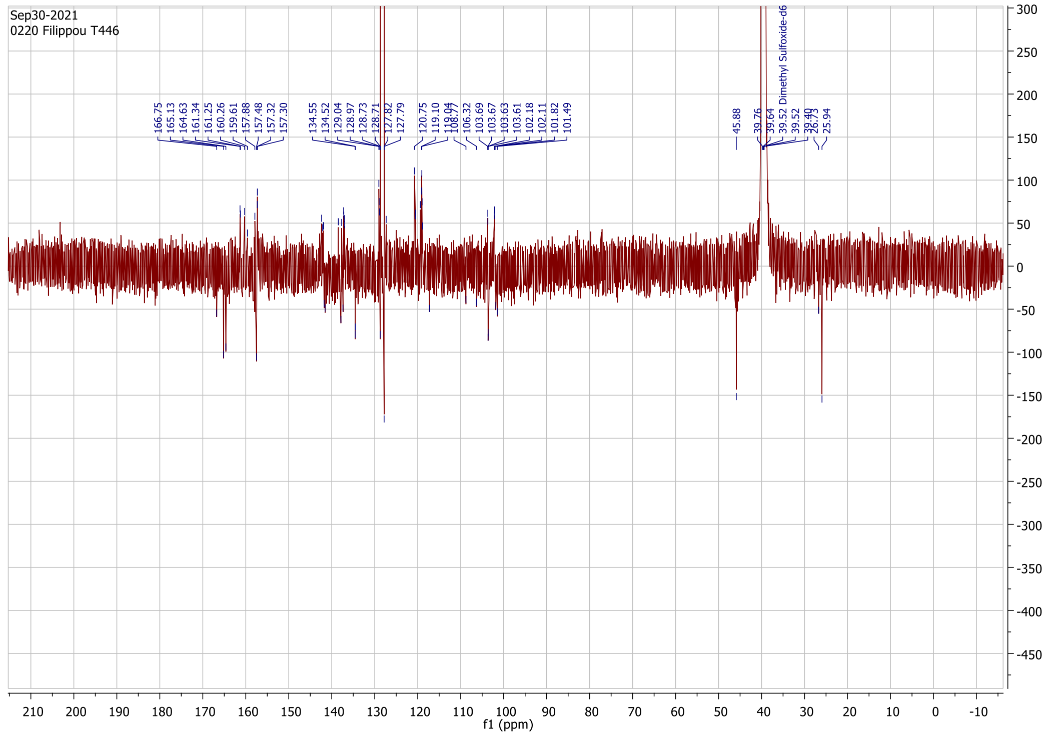

DFT-optimized complex geometries of Zr-1 – Zr-5, reacted with model TCO and corresponding cartesian xyz-coordinates

Zr-1 + TCO-butylcarbamate

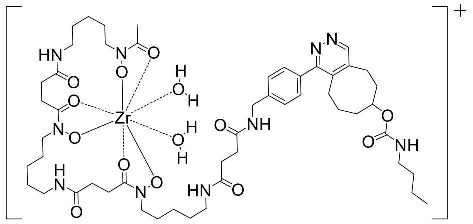

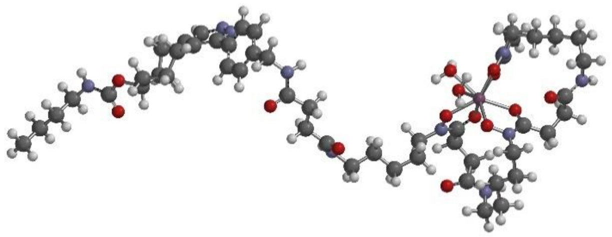

Zr	-5.4152810000	2.5526600000	-3.9809940000
C	-1.0431930000	2.0075390000	-3.6853760000
N	-2.4129580000	2.0023680000	-4.2096350000
C	-2.8846760000	1.5943490000	-5.3711440000
C	-2.0352170000	1.0299570000	-6.4796240000
C	-1.9686910000	1.9589890000	-7.7062280000
C	-1.1922590000	3.2421030000	-7.4107880000
O	-0.2303540000	3.2481800000	-6.6311490000
N	-1.6234740000	4.3421850000	-8.0686000000
C	-1.0821410000	5.6839470000	-7.8673560000
C	-2.1918820000	6.7012010000	-7.5697360000
C	-2.9959260000	6.3866580000	-6.3012050000
C	-4.2679310000	7.2370850000	-6.1895780000
C	-5.2068430000	6.8297970000	-5.0459930000
N	-5.5662410000	5.4032550000	-5.0567890000
C	-6.3276090000	4.7369780000	-5.9044930000
C	-7.0379320000	5.4133060000	-7.0477120000
C	-8.4633410000	4.8905930000	-7.2431920000
C	-9.3641650000	5.2013190000	-6.0498520000
O	-8.9053780000	5.6233990000	-4.9794420000
N	-10.6816860000	4.9668760000	-6.2529420000
C	-11.6874120000	5.0078760000	-5.1956630000
C	-12.2495000000	3.6165980000	-4.8599400000
C	-11.2040790000	2.5697480000	-4.4440380000
C	-10.4931800000	2.8682380000	-3.1166250000
C	-9.5399210000	1.7530220000	-2.6656980000
N	-8.2889820000	1.6984430000	-3.4307460000
C	0.8264610000	3.4483930000	-2.7022800000
H	1.1099760000	4.5003400000	-2.5636600000
H	1.5310410000	3.0346970000	-3.4380630000
C	0.9870290000	2.7116030000	-1.3646670000
H	0.2353920000	3.0730080000	-0.6501510000
H	0.8156950000	1.6346970000	1.4912330000
C	2.3823930000	2.9220350000	-0.7585250000
H	3.1544900000	2.5741080000	-1.4531750000
H	2.5521170000	3.9870820000	-0.5748170000
N	2.5742030000	2.2202900000	0.5062910000
H	2.8996670000	1.2631640000	0.4762980000
C	2.2410430000	2.7567820000	1.7043500000
O	1.7677670000	3.8947680000	1.8197320000
C	2.5251940000	1.8830990000	2.9218660000
H	2.7480550000	0.8522370000	2.6257100000
H	3.4223130000	2.2813850000	3.4111220000
C	1.3654020000	1.9205360000	3.9263390000
H	1.1045880000	2.9686090000	4.1143280000
H	0.4776740000	1.4326230000	3.5089230000
C	1.7665740000	1.2986360000	5.2583440000
O	2.8105390000	1.6286890000	5.8358370000
N	0.8997600000	0.3897640000	5.7682630000
H	0.1087190000	0.1024190000	5.2067850000
C	1.1364890000	-0.3322140000	7.0189670000
H	0.1771260000	-0.4388750000	7.5336420000
H	1.7843880000	0.3002910000	7.6303880000
C	1.7699260000	-1.6953880000	6.8051740000

C	-7.8946240000	0.8273140000	-4.3443800000
C	-8.7715610000	-0.2904640000	-4.8305050000
O	-3.3426210000	2.4355800000	-3.3062100000
O	-4.1612360000	1.6825180000	-5.5513440000
O	-4.9572540000	4.6317090000	-4.0986960000
O	-6.4170580000	3.4690510000	-5.7414310000
O	-7.3597670000	2.6351390000	-3.0527440000
O	-6.6977700000	0.9258800000	-4.7958040000
H	-0.3810230000	1.6154140000	-4.4553760000
H	-1.0266010000	0.8028670000	-6.1380240000
H	-2.5073230000	0.0881530000	-6.7791630000
H	-2.9753860000	2.1832610000	-8.0720490000
H	-1.4456950000	1.4243460000	-8.5088760000
H	-2.4223040000	4.2485820000	-8.6840220000
H	-0.3731450000	5.6149570000	-7.0386100000
H	-0.5225800000	5.9955890000	-8.7586520000
H	-1.7342930000	7.6962620000	-7.4971700000
H	-2.8742310000	6.7384340000	-8.4316550000
H	-2.3664840000	6.5289650000	-5.4118840000
H	-3.2771120000	5.3264950000	-6.3188930000
H	-4.0135370000	8.2941230000	-6.0367140000
H	-4.8156290000	7.1882370000	-7.1392470000
H	-6.1294640000	7.4173590000	-5.0702140000
H	-4.7374000000	6.9865600000	-4.0732670000
H	-7.0488660000	6.4952180000	-6.9060320000
H	-6.4610010000	5.2106550000	-7.9589740000
H	-8.4498250000	3.8046600000	-7.3917570000
H	-8.8882830000	5.3260540000	-8.1544740000
H	-10.9636200000	4.5939280000	-7.1519690000
H	-11.2102990000	5.4662730000	-4.3278310000
H	-12.5068800000	5.6655270000	-5.5086960000
H	-12.9930240000	3.7388310000	-4.0607000000
H	-12.7935120000	3.2387670000	-5.7360760000
H	-10.4641150000	2.4653060000	-5.2484320000
H	-11.7093610000	1.5977070000	-4.3614440000
H	-9.9335310000	3.8083700000	-3.1750540000
H	-11.2404160000	2.9891600000	-2.3212950000
C	3.1211820000	-1.8020930000	6.4446950000
C	1.0168410000	-2.8680040000	6.9338060000
C	3.7023190000	-3.0480500000	6.2216070000
H	3.7149370000	-0.8998890000	6.3276150000
C	1.5935460000	-4.1173500000	6.7025600000
H	-0.0318560000	-2.8045910000	7.2150210000
C	2.9466220000	-4.2257630000	6.3501780000
H	4.7455650000	-3.1032340000	5.9236710000
H	0.9947800000	-5.0182180000	6.7972170000
C	3.5330820000	-5.5633340000	6.0407350000
C	4.7426920000	-6.0425830000	6.6086110000
C	5.2035990000	-7.2706030000	6.1191860000
C	4.3845240000	-7.9372940000	5.1977360000
H	4.6806150000	-8.9078870000	4.8048890000
N	2.8023070000	-6.2754700000	5.1631220000
N	3.2136440000	-7.4779590000	4.7590880000
C	8.0512680000	-5.5164510000	7.8552410000
C	6.5413410000	-7.8540460000	6.4924160000
C	8.6694230000	-6.3537750000	6.7277370000
C	7.6966280000	-7.0754160000	5.7946390000
H	6.6742060000	-7.8403810000	7.5797210000
H	7.7908500000	-6.1869480000	8.6834280000
H	8.8444900000	-4.8644420000	8.2395790000
H	6.5676710000	-8.9054030000	6.1913910000
H	9.3216100000	-5.7290310000	6.1119260000
H	8.2802020000	-7.7545250000	5.1650390000
H	7.2783060000	-6.3247840000	5.1155490000
C	5.4318810000	-5.3222770000	7.7504520000
H	5.5470070000	-6.0404270000	8.5732670000
H	4.7502650000	-4.5535840000	8.1180410000
C	6.8198100000	-4.6558880000	7.4959480000
H	6.8866090000	-4.2843050000	6.4660430000
H	6.8522680000	-3.7678920000	8.1357370000
O	9.5132770000	-7.3253870000	7.4117160000
C	10.6346340000	-7.7470870000	6.7672940000
O	11.0116930000	-7.3213230000	5.6788960000
N	11.2552100000	-8.7145610000	7.4876900000

H	-10.0201060000	0.7737700000	-2.7264290000
H	-9.2318570000	1.9062870000	-1.6273820000
H	-8.5315240000	-0.4788360000	-5.8796330000
H	-8.5450000000	-1.2009340000	-4.2624050000
H	-9.8362350000	-0.0772660000	-4.7397670000
H	-1.0363850000	1.3241890000	-2.8304310000
O	-5.2598430000	3.3026950000	-1.7569250000
H	-6.1837400000	3.3183290000	-1.4391350000
H	-4.9536280000	4.2284310000	-1.7426770000
O	-5.1805640000	0.6023130000	-2.5885320000
H	-5.2963540000	-0.2276470000	-3.0848980000
H	-4.2508260000	0.6135370000	-2.2932320000
C	-0.5992220000	3.4167150000	-3.2767000000
H	-1.3024450000	3.8154860000	-2.5375880000
H	-0.6491820000	4.0557180000	-4.1645460000
H	10.9034280000	-8.8892880000	8.4205810000
C	12.5827410000	-9.2105250000	7.1413580000
H	12.6315930000	-10.2663100000	7.4303980000
H	12.6714630000	-9.1617900000	6.0526460000
C	13.7229040000	-8.4262130000	7.8038120000
H	13.6425730000	-7.3704860000	7.5105610000
H	13.5965320000	-8.4620890000	8.8951240000
C	15.1072190000	-8.9670840000	7.4253790000
H	15.2273400000	-8.9215400000	6.3337590000
H	15.1685460000	-10.0305260000	7.6968260000
C	16.2521740000	-8.2021570000	8.0977740000
H	17.2277090000	-8.6035220000	7.7996320000
H	16.2302350000	-7.1393920000	7.8266890000
H	16.1824620000	-8.2678480000	9.1906640000

Zr-2 + TCO-butylcarbamate

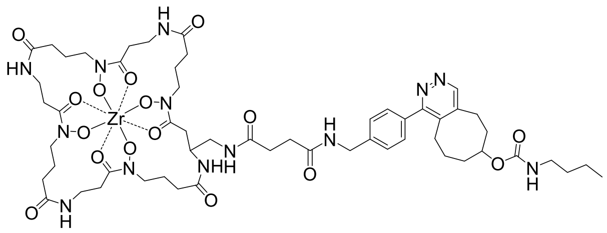

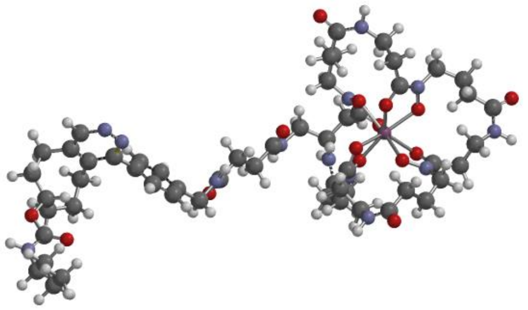

Zr	-5.7657190000	-2.2144160000	2.6742730000
O	-5.6580600000	-3.8122560000	4.2505940000
O	-6.5587690000	-4.1119700000	1.9083140000
O	-7.5941670000	-1.7043380000	3.7796840000
O	-7.4015450000	-1.6180040000	1.2563160000
O	-4.9181310000	-2.1740010000	0.5744350000
O	-3.6470760000	-2.8452330000	2.6650400000
O	-5.0428560000	-1.2691000000	4.5826900000
O	-5.2210650000	-0.1144010000	2.3359080000
C	-8.5728150000	-1.4291200000	1.7325320000
N	-8.7218860000	-1.4562920000	3.0462180000
C	-3.6642030000	-2.3948180000	0.4288790000
N	-2.9590390000	-2.7309620000	1.4923390000
C	-4.7927740000	-0.0162340000	4.5784630000
N	-4.8577640000	0.6281430000	3.4251000000
C	-5.9288200000	-5.0146000000	3.9087510000
C	-4.6401020000	2.0487650000	3.1621490000
C	-4.4925380000	0.6826490000	5.8842380000
C	-5.6523260000	-6.1366250000	4.8823810000
C	-9.9567990000	-1.3478600000	3.8181420000
C	-9.7283540000	-1.2197790000	0.7818250000
C	-1.5128140000	-2.9274740000	1.5931330000
C	-3.0542930000	-2.2068070000	-0.9402250000
C	-3.5983740000	2.2919390000	2.0599450000
C	-5.6909980000	1.4959260000	6.4252650000
O	-2.0653130000	1.9890250000	-0.5744390000
O	-3.2009100000	-6.2853830000	2.0794950000
C	-8.1912730000	-6.7597510000	1.7352270000
H	-8.9490500000	-0.8336070000	6.3021710000
H	-9.7455350000	0.6977670000	6.6137670000
H	-9.3503640000	-3.7675110000	-0.6507110000
H	-9.6092140000	-6.4968170000	0.1310720000
H	-8.1207020000	-5.6050280000	-0.1215910000
H	-2.7289030000	-5.6429400000	5.1440480000
H	-1.3199730000	-4.3518230000	3.9146530000
H	-0.5852810000	-5.8153540000	3.2853870000
N	-6.4174760000	-5.2199060000	2.6973120000
C	-6.7115250000	-6.4896790000	2.0397660000
C	-9.2070300000	0.1276990000	5.8440820000
C	-4.3441750000	-6.9105260000	4.5869270000
H	-4.1364100000	-7.5622150000	5.4415870000
H	-4.4622400000	-7.5425040000	3.7054400000
C	-2.2205530000	-0.6021300000	-2.6846120000
H	-1.7065070000	-1.4913850000	-3.0609940000
H	-3.1705970000	-0.5108140000	-3.2285910000
N	-1.3577180000	0.5431660000	-2.9326250000
H	-1.4255860000	1.3066550000	-2.2592100000
C	-0.3700770000	0.5281630000	-3.8558830000
O	-0.1694200000	-0.4251830000	-4.6209120000
C	0.4968080000	1.7816500000	-3.9112330000
H	0.3453740000	2.2446400000	-4.8931180000
H	0.1939920000	2.5095370000	-3.1509490000
C	1.9866700000	1.4335000000	-3.7625120000
H	2.2272450000	0.6275640000	-4.4657650000
H	2.1977510000	1.0646840000	-2.7531800000
C	2.8723520000	2.6226700000	-4.1124620000

O	-10.0377440000	-4.6131140000	2.3223440000
O	-7.8692620000	1.7476190000	4.6298910000
C	-10.1296230000	-0.0578350000	4.6303960000
C	-10.5726180000	-2.4953250000	0.5386500000
C	-1.0488170000	-4.3809410000	1.7457720000
C	-2.5186260000	-0.7753190000	-1.1837890000
N	-3.4826220000	0.2022200000	-0.6564170000
C	-4.1538250000	2.1701700000	0.6117390000
C	-3.1467740000	1.4583580000	-0.2717170000
N	-6.9389470000	0.7526380000	6.4575750000
C	-7.9407300000	0.9311880000	5.5552300000
N	-9.7891520000	-3.6859370000	0.2575420000
C	-8.8456050000	-5.8973900000	0.6465120000
C	-9.5820450000	-4.6690920000	1.1746510000
N	-3.1864960000	-6.0658710000	4.3468530000
C	-1.3625370000	-5.0651350000	3.0834860000
C	-2.6825850000	-5.8318030000	3.1056390000
H	-4.3026150000	2.5062410000	4.0922670000
H	-5.6070200000	2.4950080000	2.9021930000
H	-3.6260760000	1.3472330000	5.7982980000
H	-4.2304080000	-0.1063100000	6.5945190000
H	-6.3387780000	-7.2879680000	2.6811900000
H	-6.1195910000	-6.5046180000	1.1180230000
H	-6.4791050000	-6.8545110000	4.9162970000
H	-5.5853760000	-5.6675560000	5.8678280000
H	-9.9876690000	-2.2241760000	4.4749830000
H	-10.7903130000	-1.4325730000	3.1205940000
H	-9.2835420000	-0.8919770000	-0.1618570000
H	-10.3934370000	-0.4207380000	1.1262930000
H	-1.1881620000	-2.3277520000	2.4502580000
H	-1.0551860000	-2.4976590000	0.7006070000
H	-3.8543830000	-2.4197250000	-1.6566930000
H	-2.2474170000	-2.9181790000	-1.1288340000
H	-2.7679480000	1.5942030000	2.2173370000
H	-3.1866140000	3.2955970000	2.2041410000
H	-5.4480110000	1.8323840000	7.4384830000
H	-5.8653760000	2.3806440000	5.8100940000
O	2.7495500000	3.2247510000	-5.1852810000
N	3.7998860000	2.9571800000	-3.1789840000
H	3.8127510000	2.4499850000	-2.3032180000
C	4.7337290000	4.0610980000	-3.3410650000
H	4.4419580000	4.9011140000	-2.6977900000
H	4.6252920000	4.3996050000	-4.3767030000
C	6.1745160000	3.6830310000	-3.0521710000
C	7.0370900000	4.6259500000	-2.4792940000
C	6.6855980000	2.4192560000	-3.3733560000
C	8.3776070000	4.3220020000	-2.2458330000
H	6.6559760000	5.6088400000	-2.2113410000
C	8.0253280000	2.1108200000	-3.1382900000
H	6.0296220000	1.6654200000	-3.7994280000
C	8.8937320000	3.0622040000	-2.5814320000
H	9.0300360000	5.0645370000	-1.7954510000
H	8.3939750000	1.1163040000	-3.3759860000
C	10.3218520000	2.7434470000	-2.2730110000
C	11.2737730000	2.3712540000	-3.2600290000
C	12.5638930000	2.1025200000	-2.7939410000
C	12.7846740000	2.2268760000	-1.4137650000
H	13.7666620000	2.0133790000	-0.9957120000
N	10.6260770000	2.8712860000	-0.9704730000
N	11.8623780000	2.6101280000	-0.5350240000
C	14.5392770000	2.9904930000	-4.1318300000
C	13.7192990000	4.1382100000	-4.7286870000
H	15.2792400000	2.6681250000	-4.8751140000
H	15.0937190000	3.3795670000	-3.2696550000
C	12.8075820000	3.7516680000	-5.9096230000
H	13.1240470000	4.6302080000	-3.9553460000
C	11.3200020000	3.5575980000	-5.5629750000
H	12.8586320000	4.5636430000	-6.6432560000
H	13.2019500000	2.8583620000	-6.4122460000
H	10.9511800000	4.4595230000	-5.0570530000
H	10.7708770000	3.4880060000	-6.5095970000
C	13.7205670000	1.7548870000	-3.6994220000
H	14.3968120000	1.0738480000	-3.1713490000
H	13.3630280000	1.2187050000	-4.5833670000

H	-8.7784440000	-6.6832250000	2.6565250000
H	-8.2335180000	-7.8084620000	1.4188000000
H	-11.1673920000	-0.0747550000	4.9829610000
H	-10.0326520000	0.8101550000	3.9699550000
H	-11.1861890000	-2.7244170000	1.4110850000
H	-11.2443640000	-2.3010640000	-0.3037060000
H	0.0393600000	-4.3565240000	1.6155480000
H	-1.4543400000	-4.9842950000	0.9268050000
H	-1.5779350000	-0.6389990000	-0.6357430000
H	-4.3546490000	-0.1712900000	-0.2949010000
H	-5.1039770000	1.6349890000	0.6207030000
H	-4.3252050000	3.1672020000	0.1937820000
H	-7.0481590000	0.0346220000	7.1624210000
O	14.7242440000	5.0969370000	-5.1670770000
C	14.3489740000	6.4040250000	-5.2044140000
O	13.2399220000	6.8181240000	-4.8789270000
N	15.3618690000	7.1730490000	-5.6717700000
H	16.2656280000	6.7335960000	-5.7841020000
C	15.2846400000	8.6250100000	-5.6724040000
H	16.0003070000	9.0148770000	-6.3994540000
H	14.2782060000	8.9322610000	-5.9637230000
C	10.9263050000	2.3092280000	-4.7338090000
H	11.3876660000	1.4222990000	-5.1837280000
H	9.8492440000	2.1787160000	-4.8393490000
H	15.5102550000	9.0548170000	-4.6872940000

Zr-3 + TCO-methylcarbamate

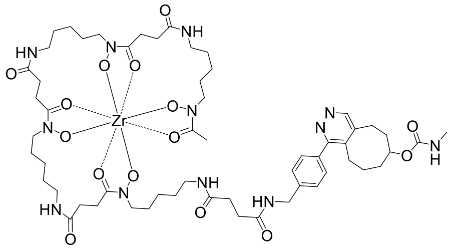

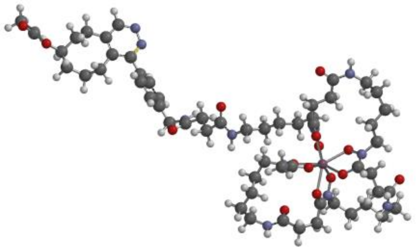

Zr	-5.8027050000	1.4392630000	-3.3911070000
C	-8.2876350000	5.1595530000	-3.5308370000
N	-7.8577400000	3.7700410000	-3.7372940000
C	-8.1603070000	2.9524330000	-4.7301150000
C	-9.2429960000	3.2472740000	-5.7402980000
C	-10.4879740000	2.3584120000	-5.5491220000
C	-11.1739630000	2.6218290000	-4.2092560000
O	-11.2891980000	3.7675620000	-3.7533980000
N	-11.6362940000	1.5235620000	-3.5700830000
C	-12.2284830000	1.5371600000	-2.2344490000
C	-11.7177510000	0.3578370000	-1.3969470000
C	-10.2021200000	0.3880540000	-1.1564470000
C	-9.6598880000	-0.9423600000	-0.6218980000
C	-8.1289640000	-0.9982700000	-0.5120320000
N	-7.4275070000	-0.5913980000	-1.7365650000
C	-7.3023340000	-1.2370110000	-2.8858840000
C	-7.9051730000	-2.6085170000	-3.0810050000
C	-7.0216210000	-3.5291210000	-3.9244480000
C	-5.6852450000	-3.8364410000	-3.2517820000
O	-5.3396240000	-3.2879290000	-2.1967560000
N	-4.9169530000	-4.7425130000	-3.9027940000
C	-3.5396950000	-5.0552150000	-3.5358540000
C	-2.5337730000	-4.6350260000	-4.6187680000
C	-2.5600040000	-3.1452780000	-4.9923860000
C	-2.1169540000	-2.1972160000	-3.8700480000
C	-2.0477620000	-0.7272010000	-4.3049340000
N	-3.3636200000	-0.1019900000	-4.4584950000
C	-3.9686770000	0.3018330000	-5.5633540000
C	-3.3848570000	0.0785260000	-6.9324750000
C	-3.5381360000	5.9734230000	-3.0724490000
H	-3.8113930000	6.1025460000	-2.0181100000
H	-4.4738720000	5.8052760000	-3.6107160000
C	-2.6738510000	4.7141730000	-3.2356960000
H	-1.7974580000	4.7420820000	-2.5853630000
H	-2.3206500000	4.6265730000	-4.2659260000
C	-3.5788320000	3.5276390000	-2.9642860000
O	-4.2376210000	3.0144350000	-3.9314820000
N	-3.8214890000	3.0932060000	-1.7370350000
C	-3.2274450000	3.5330030000	-0.4749860000
H	-4.0372550000	3.9511570000	0.1340280000
H	-2.5194640000	4.3342590000	-0.6912810000
O	-4.8105230000	2.1599020000	-1.5831650000
C	-2.5310630000	2.3973990000	0.2840560000
H	-3.2728110000	1.6352810000	0.5486680000
H	-2.1640120000	2.8228810000	1.2275130000
C	-1.3707420000	1.7518030000	-0.4817410000
H	-1.7522960000	1.3066970000	-1.4100940000
H	-0.6479060000	2.5249300000	-0.7804490000
C	-0.6521060000	0.6706530000	0.3343860000
H	-0.1898060000	1.1158130000	1.2257370000
H	-1.3824720000	-0.0689660000	0.6920570000
C	0.4301180000	-0.0478400000	-0.4820750000
H	1.1813710000	0.6658010000	-0.8306470000
H	-0.0185150000	-0.5205100000	-1.3642810000
N	1.1340900000	-1.0742570000	0.2774800000
H	0.6488750000	-1.9439420000	0.4563680000
C	2.3568680000	-0.8891910000	0.8322460000
O	2.9941460000	0.1657750000	0.7186010000

O	-6.8395990000	3.3622670000	-2.9223380000	C	2.9251530000	-2.0937740000	1.5764590000
O	-7.5055630000	1.8515680000	-4.8079510000	H	2.1319340000	-2.8033560000	1.8355310000
O	-6.8849380000	0.6684250000	-1.7040170000	H	3.6069730000	-2.6147950000	0.8932050000
O	-6.6901660000	-0.6469140000	-3.8336890000	C	3.7088700000	-1.6732580000	2.8212990000
O	-3.9811830000	0.1970700000	-3.2727370000	H	4.3888100000	-0.8569800000	2.5457390000
O	-5.0788710000	0.9282620000	-5.4542770000	H	3.0367580000	-1.2780500000	3.5912980000
H	-9.2792160000	5.2861480000	-3.9653540000	C	4.5591050000	-2.8086740000	3.3802870000
H	-8.3781020000	5.2909280000	-2.4482230000	O	5.0842890000	-3.6579150000	2.6495680000
H	-9.5378680000	4.2960550000	-5.7162300000	N	4.7118840000	-2.8041330000	4.7284630000
H	-8.8193070000	3.0449410000	-6.7295250000	H	4.3076920000	-2.0431870000	5.2595590000
H	-10.2201900000	1.3025160000	-5.6543130000	C	5.5822400000	-3.7373310000	5.4415750000
H	-11.2018410000	2.5939350000	-6.3480570000	H	5.6865430000	-4.6166250000	4.8006260000
H	-11.5125000000	0.6225490000	-4.0148970000	H	5.0737290000	-4.0451600000	6.3602520000
H	-11.9623310000	2.4945020000	-1.7783070000	C	6.9443020000	-3.1537780000	5.7741680000
H	-13.3235110000	1.4982620000	-2.3064130000	C	7.8579450000	-2.8504350000	4.7540210000
H	-12.2594170000	0.3535340000	-0.4425660000	C	7.3088320000	-2.8902580000	7.0987780000
H	-11.9884200000	-0.5773310000	-1.9088550000	C	9.1041330000	-2.3046170000	5.0529940000
H	-9.9498560000	1.2054600000	-0.4665820000	H	7.5865640000	-3.0403770000	3.7191370000
H	-9.6997300000	0.6136790000	-2.1041800000	C	8.5551020000	-2.3389240000	7.4018450000
H	-10.0672710000	-1.1539560000	0.3756100000	H	6.6124870000	-3.1175780000	7.9028220000
H	-10.0042610000	-1.7556920000	-1.2737860000	C	9.4731610000	-2.0481780000	6.3842770000
H	-7.7975900000	-2.0039740000	-0.2339750000	H	9.7903220000	-2.0627340000	4.2456570000
H	-7.7657680000	-0.3081770000	0.2519340000	H	8.8205620000	-2.1342590000	8.4349790000
H	-8.1205760000	-3.0733680000	-2.1172050000	C	10.7800740000	-1.4058210000	6.7255620000
H	-8.8655530000	-2.4772060000	-3.5962220000	C	12.0457390000	-1.9639430000	6.3993370000
H	-6.8241190000	-3.0744280000	-4.9018180000	C	13.1593560000	-1.1946470000	6.7562000000
H	-7.5560920000	-4.4663060000	-4.1191250000	C	12.9124590000	0.0234580000	7.4067730000
H	-5.2672970000	-5.1264580000	-4.7725060000	H	13.7430020000	0.6643740000	7.6963200000
H	-3.3483260000	-4.5446620000	-2.5904950000	N	10.6345370000	-0.2430280000	7.3853480000
H	-3.4496580000	-6.1332630000	-3.3544520000	N	11.7040320000	0.4768270000	7.7302590000
H	-1.5299230000	-4.9145910000	-4.2703130000	C	12.1723820000	-3.3093680000	5.7120110000
H	-2.7245460000	-5.2298110000	-5.5225710000	C	15.0284690000	-2.6311120000	7.6770190000
H	-3.5702970000	-2.8744700000	-5.3269630000	C	12.8691720000	-4.4640440000	6.4972800000
H	-1.8991320000	-2.9978650000	-5.8578320000	C	15.3570580000	-4.0487680000	7.2006770000
H	-2.7874830000	-2.2717450000	-3.0066320000	C	14.3718390000	-4.6676450000	6.2002040000
H	-1.1148960000	-2.4835560000	-3.5232660000	H	12.6987670000	-3.1697540000	4.7582920000
H	-1.4981700000	-0.6223800000	-5.2432540000	H	12.6878380000	-4.3591740000	7.5737340000

H	-1.5267940000	-0.1314960000	-3.5483900000
H	-4.2111240000	-0.0380020000	-7.6381530000
H	-2.8051160000	0.9593690000	-7.2349260000
H	-2.7418390000	-0.7996930000	-6.9937430000
C	-7.2417890000	6.1130020000	-4.1157680000
H	-6.2856150000	5.8108780000	-3.6852000000
H	-7.1679890000	5.9380270000	-5.1976420000
C	-7.4609060000	7.6114340000	-3.8653780000
H	-7.7276350000	7.7891640000	-2.8137980000
H	-8.3095420000	7.9651150000	-4.4638380000
C	-6.1922290000	8.4181600000	-4.2264600000
H	-6.4570620000	9.4525660000	-4.4737530000
H	-5.7416250000	7.9877770000	-5.1300320000
C	-5.1377960000	8.4194610000	-3.0872350000
H	-5.2542850000	9.3226560000	-2.4791850000
H	-5.3132400000	7.5788280000	-2.4144120000
N	-3.7428290000	8.3623160000	-3.5311040000
H	-3.3361010000	9.2102420000	-3.9111780000
C	-2.9314920000	7.2697070000	-3.5902260000
O	-1.7796100000	7.3348750000	-4.0333420000

H	14.2377110000	-2.7140850000	8.4302120000
H	15.4172190000	-4.7085540000	8.0742160000
H	11.1715150000	-3.6529270000	5.4531600000
H	15.9002690000	-2.2323360000	8.2047030000
H	12.3553900000	-5.3835860000	6.1990550000
H	14.5934080000	-5.7397990000	6.1572870000
H	14.6011970000	-4.2744330000	5.2030830000
C	14.5883000000	-1.6369640000	6.5614210000
H	14.7234980000	-2.0835220000	5.5711220000
H	15.2319820000	-0.7527170000	6.5897190000
O	16.6353260000	-4.0735350000	6.4990600000
C	17.8464440000	-4.0289840000	7.1177470000
O	18.8500840000	-3.9379090000	6.4179600000
N	17.8850120000	-4.0775520000	8.4720310000
H	17.0546730000	-4.2891170000	9.0045550000
C	19.1631710000	-4.1320230000	9.1704720000
H	18.9665620000	-4.0880930000	10.2431280000
H	19.7863880000	-3.2791410000	8.8878200000
H	19.7119880000	-5.0538940000	8.9443090000

Zr-4 + TCO-butylcarbamate

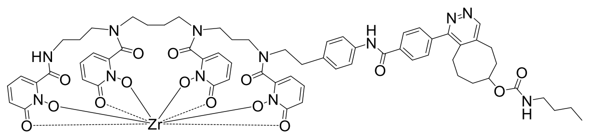

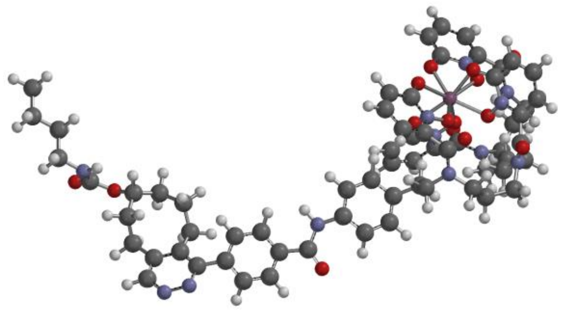

C	-1.8255460000	-0.1863970000	-2.3779470000	H	-6.5525150000	3.0450480000	-0.3698830000
C	-1.6976300000	-0.2465180000	-1.0018340000	C	-7.2242010000	2.3494990000	-3.5028090000
C	-2.7891900000	0.1035390000	-0.1812220000	H	-7.0198940000	2.0548080000	-4.5392360000
H	-0.9872230000	-0.4614160000	-3.0104030000	H	-7.3492940000	3.4350330000	-3.4905500000
H	-0.7813240000	-0.5650480000	-0.5197610000	C	-8.5426540000	1.6835590000	-3.0726100000
O	-4.9493800000	0.8683130000	0.0147690000	H	-8.4928810000	0.6072970000	-3.2567500000
O	-2.8438410000	0.0575880000	1.0995030000	H	-9.3037120000	2.0857420000	-3.7539140000
C	-5.4716810000	0.8353220000	-2.7710490000	Zr	-4.7722760000	0.4692370000	2.1886830000
O	-5.9976330000	-0.1081530000	-3.3728100000	C	-3.0394290000	0.2050590000	-2.9646600000
N	-6.0113500000	2.0716640000	-2.7048630000	H	-3.1697350000	0.2174960000	-4.0401210000
C	-5.3108000000	3.2203690000	-2.1134410000	N	-3.9380020000	0.5349190000	-0.8088460000
H	-4.3301870000	2.8827130000	-1.7756740000	C	-4.1023350000	0.5739090000	-2.1594820000
H	-5.1354550000	3.9621110000	-2.9022420000	C	-0.2130590000	3.8287000000	2.6392910000
C	-1.4590590000	2.0451070000	4.5563120000	H	0.3869130000	4.7148600000	2.4081060000
C	-0.4456810000	1.4605930000	5.2917650000	H	0.4188990000	3.1678310000	3.2368050000
C	-0.4247140000	0.0611350000	5.4291150000	C	-0.5943640000	3.0997520000	1.3296870000
C	-1.4207750000	-0.7233940000	4.8719830000	H	-1.0547090000	3.8048840000	0.6305410000
C	-2.4641820000	-0.1198840000	4.1366590000	H	-1.3377530000	2.3276020000	1.5516310000
N	-2.3992400000	1.2466120000	3.9854290000	C	0.6332480000	2.4803800000	0.6976560000
H	0.3747140000	-0.4085680000	5.9940690000	C	1.4300170000	3.1997090000	-0.2007830000
H	0.3066030000	2.0874690000	5.7561420000	C	1.0423190000	1.1811140000	1.0351900000
H	-1.4365550000	-1.8013530000	4.9866880000	C	2.5937110000	2.6627340000	-0.7538690000
O	-3.3996720000	1.8059160000	3.2788010000	H	1.1354310000	4.2076880000	-0.4850440000
O	-3.4742430000	-0.6993080000	3.5985550000	C	2.1972450000	0.6263180000	0.4928290000
C	-1.8007910000	3.5257740000	4.5088250000	H	0.4484670000	0.5932710000	1.7318050000
O	-2.5416970000	3.9298480000	5.4084040000	C	2.9898430000	1.3621650000	-0.4049380000
N	-1.3325800000	4.2790260000	3.4842080000	H	3.1894870000	3.2397420000	-1.4460880000
C	-1.8459690000	5.6527560000	3.3318550000	H	2.4941350000	-0.3835420000	0.7672470000

H	-2.5186650000	5.8226000000	4.1701200000
H	-1.0002210000	6.3424890000	3.4316420000
C	-6.3141860000	4.1065520000	3.9953330000
C	-7.1327400000	4.3240770000	5.0942170000
C	-7.7202730000	3.2502450000	5.7734960000
C	-7.3910440000	1.9547400000	5.4211700000
C	-6.5348030000	1.7210300000	4.3270200000
N	-6.1566010000	2.8138640000	3.5583890000
H	-8.3813910000	3.4362180000	6.6141280000
H	-7.2422730000	5.3399900000	5.4513160000
H	-7.7507070000	1.0920200000	5.9705380000
O	-5.5898630000	2.5138230000	2.3695620000
O	-6.0820650000	0.5773150000	3.9652730000
C	-5.3807950000	5.2524970000	3.6043240000
O	-4.9021980000	5.8633460000	4.5684820000
N	-5.0627100000	5.5472870000	2.3248720000
C	-3.9885100000	6.5357250000	2.0923340000
H	-4.2279390000	7.0406070000	1.1533220000
H	-4.0247850000	7.2830780000	2.8892830000
C	-8.3410300000	-1.3158770000	0.2397680000
C	-8.6778270000	-2.6378940000	-0.0054080000
C	-7.8648570000	-3.6740950000	0.4693210000
C	-6.6974220000	-3.3758530000	1.1497690000
C	-6.3544870000	-2.0343670000	1.3972760000
N	-7.2325080000	-1.0511270000	0.9957930000
H	-8.1336860000	-4.7076350000	0.2755690000
H	-9.5606090000	-2.8280860000	-0.6015950000
H	-6.0158540000	-4.1430260000	1.4996340000
O	-6.9372390000	0.1906480000	1.4080040000
O	-5.2781350000	-1.6346870000	1.9851670000
C	-9.1223810000	-0.2373440000	-0.4782280000
O	-10.2305610000	-0.5213200000	-0.9484520000
N	-8.5039810000	0.9454850000	-0.6729500000
H	-7.6031980000	1.0859940000	-0.2227990000
C	-9.0250900000	1.9208960000	-1.6273500000
H	-8.7557080000	2.9204740000	-1.2759360000
H	-10.1136180000	1.8419270000	-1.5980730000
N	4.1449830000	0.7253670000	-0.9093040000
H	4.2092550000	-0.2582450000	-0.6818220000
C	5.1838040000	1.2660240000	-1.6184910000
O	5.2011230000	2.4377300000	-2.0083120000
C	6.3343720000	0.3415300000	-1.9129840000
C	6.5639100000	-0.8749900000	-1.2522590000
C	7.2532010000	0.7607780000	-2.8869890000
C	7.6713190000	-1.6574000000	-1.5738800000
H	5.9108240000	-1.2213090000	-0.4565810000
C	8.3640710000	-0.0148190000	-3.2001710000
H	7.0851190000	1.7067220000	-3.3904790000
C	8.5848150000	-1.2407380000	-2.5536930000
H	7.8386870000	-2.5851760000	-1.0344470000
H	9.0682430000	0.3260040000	-3.9529230000
C	9.8171180000	-2.0302970000	-2.8495520000
C	9.8012630000	-3.4009100000	-3.2144540000
C	11.0548890000	-4.0082510000	-3.3514210000
C	12.1808770000	-3.1875810000	-3.1976890000
H	13.1795160000	-3.6026560000	-3.3181930000
C	11.2340230000	-5.4832910000	-3.6025740000
C	10.9374830000	-6.3083670000	-2.3147520000
C	8.5120020000	-4.1284270000	-3.5379700000
C	9.7459550000	-7.2657680000	-2.4060050000
C	8.0403430000	-5.2775000000	-2.5961710000
C	8.4585190000	-6.7017590000	-3.0229990000
H	10.5858990000	-5.8108570000	-4.4227460000
H	10.7479310000	-5.6313210000	-1.4746010000
H	8.6079760000	-4.5377850000	-4.5523920000
H	6.9456590000	-5.2498450000	-2.6061510000
H	12.2616400000	-5.6652950000	-3.9295280000
H	11.8228120000	-6.8872710000	-2.0332750000
H	7.7137570000	-3.3859180000	-3.5937410000
H	9.5096080000	-7.6254390000	-1.3977110000
H	8.3326690000	-5.0732210000	-1.5593930000
H	7.6548550000	-7.4033070000	-2.7725350000
H	8.5569130000	-6.7378180000	-4.1144590000
O	10.0681730000	-8.4062200000	-3.2558600000

C	-2.5794030000	5.9103640000	1.9831950000
H	-2.6652730000	4.9744630000	1.4280670000
H	-1.9686710000	6.5810080000	1.3678400000
C	-5.9114610000	5.1946140000	1.1823240000
H	-6.6662070000	4.4887920000	1.5310970000
H	-6.4384290000	6.1018110000	0.8544870000
C	-5.1339040000	4.5859870000	0.0128950000
H	-4.4066790000	3.8714960000	0.4092740000
H	-4.5758360000	5.3634200000	-0.5251190000
C	-6.0711880000	3.8449690000	-0.9378340000
H	-6.8600040000	4.5148880000	-1.3031730000
C	10.8337380000	-9.4616650000	-2.8669790000
O	11.1244960000	-10.3047020000	-3.7093490000
N	11.2464280000	-9.5236180000	-1.5767270000
H	10.8616780000	-8.8983070000	-0.8848030000
C	11.9660800000	-10.6947130000	-1.0910990000
H	12.3072990000	-10.4880640000	-0.0752760000
H	12.8334660000	-10.8926120000	-1.7258580000
N	10.9469010000	-1.3101140000	-2.7331120000
N	12.1343490000	-1.8835800000	-2.9287560000
H	11.3311550000	-11.5890610000	-1.0806440000

Zr-5 + TCO-butylcarbamate

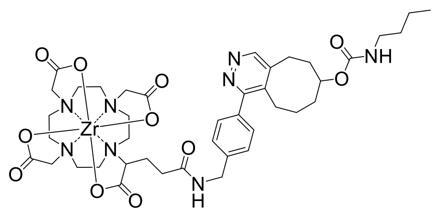

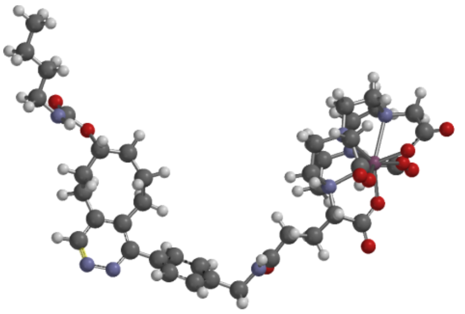

Zr	-4.6427840000	0.6087830000	5.3787560000	O	2.4088720000	-1.9242700000	4.4574390000
O	-4.9952930000	1.7796360000	7.1661000000	C	3.2039730000	-4.2454710000	3.0319830000
O	-4.2408870000	3.2422750000	8.6920570000	H	3.1474160000	-5.3353780000	3.1091620000
O	-8.7076760000	0.4541270000	6.5263390000	H	3.7667440000	-3.8701670000	3.8900370000
O	-6.6469000000	0.0235770000	5.7507520000	N	1.8488160000	-3.7088370000	3.1548090000
O	-4.7367610000	-1.4428890000	4.7114690000	H	1.1191730000	-4.1493570000	2.6091290000
O	-5.4548190000	-2.9806600000	3.2426150000	C	3.8863080000	-3.8490500000	1.7349800000
N	-2.3407900000	0.0496580000	4.3947890000	C	4.0757470000	-4.7786650000	0.7067350000
N	-3.3481780000	2.7124810000	5.2000780000	C	4.3271050000	-2.5320510000	1.5384620000
N	-6.2631110000	2.4411810000	4.7546360000	H	3.7449790000	-5.8056890000	0.8434060000
N	-4.8929390000	0.5973110000	2.9153370000	C	4.9291390000	-2.1539200000	0.3406220000
C	-1.4229070000	1.1968010000	4.6714510000	H	4.1998370000	-1.8031370000	2.3342400000
C	-2.0561020000	2.5613330000	4.4548510000	C	5.1082250000	-3.0845360000	-0.6967480000
C	-4.1511250000	3.8084900000	4.5579490000	H	5.2812020000	-1.1332830000	0.2172000000
C	-5.6291380000	3.7832120000	4.9294550000	C	5.8104710000	-2.7146580000	-1.9634490000
C	-6.7676700000	2.1877160000	3.3692080000	C	5.4008040000	-1.6526960000	-2.8152550000
C	-5.6851260000	1.7617020000	2.3945880000	C	6.2320030000	-1.3998820000	-3.9131820000
C	-3.5574820000	0.5798930000	2.2331310000	C	7.3447470000	-2.2379300000	-4.0790100000
C	-2.4966190000	-0.2566040000	2.9394830000	H	8.0239770000	-2.0820910000	-4.9147820000
C	-3.0485150000	3.0614070000	6.6266560000	N	6.8784180000	-3.4922640000	-2.2166930000
C	-4.1761130000	2.7009050000	7.5933880000	N	7.6456940000	-3.2613480000	-3.2836540000
C	-7.4190020000	2.2912050000	5.6785470000	C	4.1400850000	-0.8524030000	-2.5438540000
C	-7.6586170000	0.8225840000	6.0179100000	C	4.9098140000	-0.8146450000	-5.9961650000
C	-5.6340910000	-0.6728580000	2.6333920000	C	3.0019620000	-0.8942230000	-3.6109810000
C	-5.2472800000	-1.8174820000	3.5693600000	C	3.6093480000	-0.0100200000	-6.0056470000
C	-1.8690250000	-1.1635540000	5.1543250000	C	2.9836460000	0.2665970000	-4.6315910000
H	-0.5220720000	1.1336270000	4.0490410000	H	4.4175120000	0.1958860000	-2.3685250000
H	-1.0973350000	1.1087020000	5.7106880000	H	2.9886840000	-1.8677070000	-4.1154280000

H	-1.3489740000	3.3367360000	4.7761720000
H	-2.2472020000	2.7382860000	3.3982310000
H	-4.0271380000	3.7261600000	3.4793750000
H	-3.7342550000	4.7822130000	4.8452550000
H	-5.7538590000	4.0745060000	5.9728870000
H	-6.1521550000	4.5358280000	4.3257280000
H	-7.2722780000	3.0822950000	2.9820260000
H	-7.5246910000	1.4028600000	3.4405900000
H	-6.1454650000	1.4999570000	1.4339080000
H	-4.9974380000	2.5818220000	2.1963690000
H	-5.4930880000	-0.9705640000	1.5887930000
H	-6.7034070000	-0.4966990000	2.7845600000
H	-3.2225180000	1.6112230000	2.1400830000
H	-3.6752520000	0.1977450000	1.2108000000
H	-2.4509650000	-2.0031730000	4.7626370000
H	-2.7339790000	-1.3172400000	2.8474820000
H	-1.5541170000	-0.0948130000	2.4029930000
H	-2.8012690000	4.1248900000	6.7125390000
H	-2.1709680000	2.4936940000	6.9482250000
H	-8.3283000000	2.7384860000	5.2615620000
H	-7.1986320000	2.7949010000	6.6210200000
C	-2.3385680000	-1.0435750000	6.6193780000
O	-3.3452900000	-0.2211570000	6.8220100000
O	-1.8316530000	-1.7102920000	7.5110950000
C	-0.3765180000	-1.5378830000	5.0806640000
H	-0.2401610000	-2.3005780000	5.8530340000
H	0.2600490000	-0.6983180000	5.3716970000
C	0.1072330000	-2.1064540000	3.7392480000
H	-0.5409530000	-2.9272390000	3.4105930000
H	0.0782920000	-1.3430270000	2.9525010000
C	1.5597070000	-2.5664500000	3.8266260000
H	4.6484510000	-1.8623000000	-5.8145740000
H	2.8708890000	-0.5370210000	-6.6212680000
H	3.7124670000	-1.2067510000	-1.6063780000
H	5.3436820000	-0.7858960000	-7.0006990000
H	2.0633160000	-0.8412430000	-3.0499780000
H	1.9479190000	0.5773770000	-4.8086920000
H	3.4856960000	1.1389790000	-4.1971450000
C	5.9606250000	-0.3398780000	-4.9502690000
H	5.6302320000	0.5874570000	-4.4715850000
H	6.8997630000	-0.1038280000	-5.4596090000
C	4.6851080000	-4.4049270000	-0.4921550000
H	4.8305510000	-5.1397390000	-1.2785740000
O	3.8341470000	1.3194320000	-6.5624810000
C	3.9897290000	1.5821480000	-7.8885850000
O	4.3278160000	2.7168320000	-8.2115600000
N	3.7801440000	0.5742190000	-8.7740590000
H	3.3335740000	-0.2778910000	-8.4661590000
C	3.7820090000	0.8308180000	-10.2153220000
H	3.9825580000	-0.1243110000	-10.7115100000
H	4.6198490000	1.4998070000	-10.4305270000
C	2.4719540000	1.4360010000	-10.7353360000
H	2.2882280000	2.3827910000	-10.2108910000
H	1.6399370000	0.7640070000	-10.4823520000
C	2.5003390000	1.6781720000	-12.2494830000
H	3.3500370000	2.3303370000	-12.4966720000
H	2.6838080000	0.7254160000	-12.7661240000
C	1.2049010000	2.3066290000	-12.7743270000
H	0.3414330000	1.6620220000	-12.5680170000
H	1.0177650000	3.2759610000	-12.2960450000
H	1.2505460000	2.4698760000	-13.8572980000

Results of radio-iTLC analyses of [^{89}Zr]Zr-31 and [^{89}Zr]Zr-32

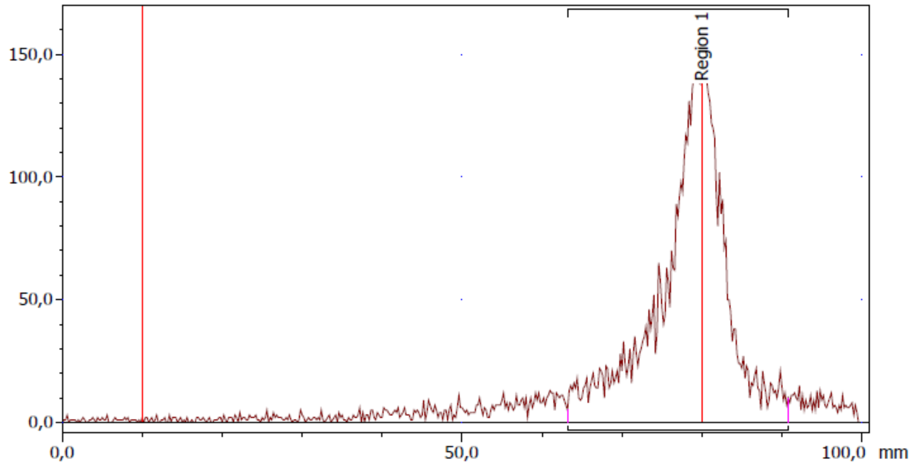

$^{89}ZrCl_4$

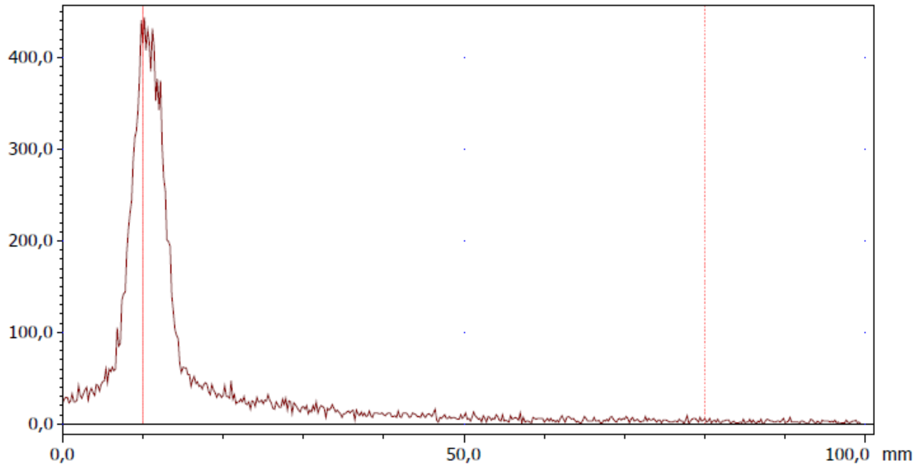

[^{89}Zr]Zr-**31**

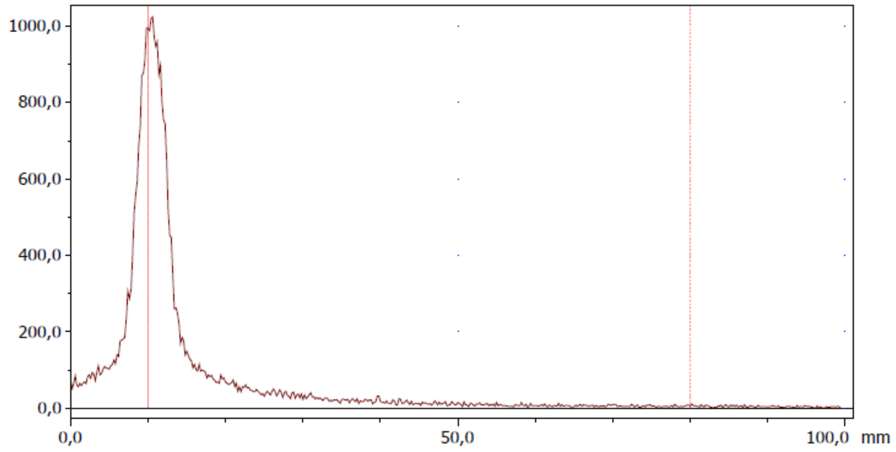

[^{89}Zr]Zr-**32**
